# WTAP-mediated m^6^A methylation of circRNA_404908 promotes esophageal squamous cell carcinoma progression

**DOI:** 10.1016/j.jbc.2025.110512

**Published:** 2025-07-22

**Authors:** Yingjie Pan, Hang Yang, Jiayi Zhang, Ruolan Zhang, Mi Yang, Qiaoling Chen, Jun Bie, Kang Liu, Guiqin Song

**Affiliations:** 1 Nanchong Key Laboratory of Cancer Biotherapy, The Second Clinical College of North Sichuan Medical College, Beijing Anzhen Nanchong Hospital of Capital Medical University & Nanchong Central Hospital, Nanchong, Sichuan, China; 2 Institute of Basic Medicine and Forensic Medicine, North Sichuan Medical College, Nanchong, Sichuan, China; 3 Department of Laboratory Medicine, Chongqing University Fuling Hospital, School of Medicine, Chongqing University, Chongqing, China

**Keywords:** ANO1, circRNA_404908, ESCC, m^6^A, miR-3059-5p, WTAP

## Abstract

N6-methyladenosine (m^6^A) RNA methylation and circular RNA have been demonstrated to exert a crucial role in diverse malignant tumors, such as esophageal squamous cell carcinoma (ESCC). Nevertheless, the precise regulatory mechanism through which m^6^A-modified circRNA impacts ESCC remains to be elucidated. Herein, we discovered that the methyltransferase Wilms’ tumor 1-associated protein (WTAP) is highly expressed in ESCC and is correlated with a poor prognosis. Knockdown of WTAP significantly diminishes the proliferation, migration, and invasion capabilities of ESCC cells both *in vitro* and *in vivo*. The m^6^A-circRNA epitranscriptomic microarray analysis, MeRIP-qPCR, RT-qPCR, and circularization verification ascertained that circRNA_404908 is the downstream target of WTAP. Knockdown of WTAP reduces the m^6^A level, expression, and stability of circRNA_404908. A series of functional assays indicate that circRNA_404908 facilitates the proliferation, migration, and invasion of ESCC cells, and overexpression of circRNA_404908 can counteract the reduction in cell proliferation, migration, and invasion abilities caused by si-WTAP. In addition, *in vitro* experiments demonstrated that circRNA_404908 regulates the expression of ANO1 by sponging miR-3059-5p, thereby promoting the progression of ESCC. Mechanistically, WTAP-mediated m^6^A modification of circRNA_404908 governs the miR-3059-5p/ANO1 axis to facilitate the advancement of ESCC. Collectively, our study reveals that WTAP-mediated m^6^A modification drives ESCC progression *via* circRNA_404908/miR-3059-5p/ANO1 axis, providing both mechanistic insights into m^6^A-circRNA crosstalk and potential therapeutic targets for ESCC treatment.

Esophageal cancer (EC) represents the seventh leading cause of cancer-related mortality globally. Recent global estimates reveal over 500,000 newly diagnosed cases and more than 400,000 deaths attributed to EC in 2022 (1). Esophageal squamous cell carcinoma (ESCC) constitutes the predominant subtype of esophageal cancer, comprising roughly 90% of cases globally (2). Currently, ESCC is primarily treated with surgery and chemoradiotherapy, but the outcomes remain suboptimal, with 5-year survival rates below 20% (3, 4). Identifying therapeutic targets and elucidating the molecular mechanisms underlying ESCC is therefore of critical importance.

N6-methyladenosine (m^6^A) represents the most abundant chemical modification in mRNA (5, 6). The m^6^A enzyme system consists of recognition proteins, demethylases, and methyltransferases, among others. Wilms tumor 1 associated protein (WTAP) is a key regulatory subunit of the m^6^A methyltransferase complex, it recruits m^6^A methyltransferase complex to target mRNAs and regulates biological processes such as RNA splicing, cell proliferation, and tumorigenesis (7, 8, 9). Recent studies have revealed that WTAP-mediated m^6^A modification of lncRNA Snhg1 modulates cardiomyocyte apoptosis and mitochondrial reactive oxygen species production, driving the progression of myocardial ischemia-reperfusion injury *via* the miR-361-5p/OPA1 axis (10). Furthermore, WTAP enhances gastric cancer development by mediating the m^6^A modification of FAM83H-AS1 and promotes the progression of laryngeal squamous cell carcinoma by stabilizing PLAU (11, 12). However, WTAP-mediated m^6^A modification in ESCC remains largely unknown.

In recent years, circular RNAs (circRNAs) have emerged as a prominent class of noncoding RNAs, playing pivotal roles in cancer development and progression through various mechanisms. The circRNAs hold significant potential as diagnostic, prognostic, and predictive biomarkers (13, 14, 15). An increasing body of evidence underscores the involvement of circRNAs in the regulation of ESCC tumorigenesis, with a predominant focus on their role as microRNA (miRNA) sponges as a potential mechanism (16, 17). For instance, the oncogenic hsa_circ_0001165, mediated by EIF4A3, promotes ESCC progression through the miR-381-3p/TNS3 pathway (18). Similarly, circ-TTC17 enhances ESCC cell growth and metastasis and suppresses autophagy-mediated radiosensitivity *via* the miR-145-5p/SIRT1 axis (19). In addition, circRNA_101491 regulates ESCC radiosensitivity by sponging miR-125a-5p (20). However, the role of m^6^A modification in circRNAs within the pathogenesis of ESCC remains to be further explored.

In our recent study, we observed that WTAP expression is upregulated in ESCC tissues and cells, where it promotes oncogenic activity both *in vitro* and *in vivo*. WTAP regulates the expression of circRNA_404908 by mediating its m^6^A modification, thereby enhancing ESCC cell growth, migration, and invasion through the miR-3059-5p/ANO1 axis. These findings highlight that WTAP and circRNA_404908 can serve as potential therapeutic targets for ESCC.

## Results

### WTAP is highly expressed in ESCC and correlates with poor prognosis

**Figure 1:**
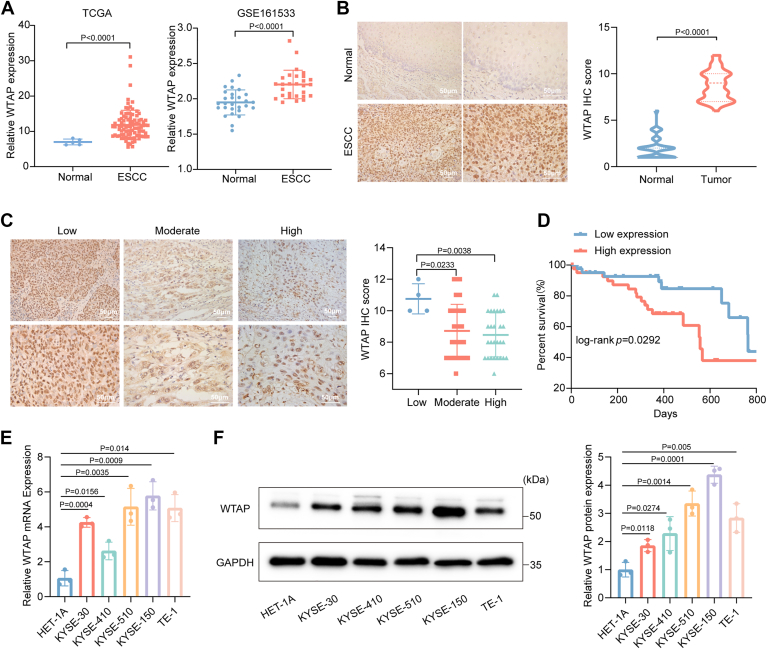
**WTAP is highly expressed in ESCC tissues and cells and is associated with poor prognosis.***A*, analysis of WTAP expression levels based on the TCGA (*left*) and GEO (*right*) databases. The TCGA dataset includes five normal tissues and 93 ESCC tissues, while the GEO dataset comprises 28 paired ESCC tissues and their corresponding normal tissues (GSE161533), *p* < 0.0001. *B*, IHC analysis of WTAP expression levels in ESCC patient tissues (n = 71), *p* < 0.0001. *C*, correlation between WTAP expression levels and the differentiation status of ESCC tissues, *p* < 0.05. *D*, Kaplan–Meier analysis of the relationship between WTAP expression levels and the prognosis of ESCC patients, *p* < 0.05. *E* and *F*, RT-qPCR and western blot analyses of WTAP expression levels in the normal esophageal epithelial cell line (HET-1A) and ESCC cell lines (n = 3), *p* < 0.05. The differences between the two groups were investigated using Student’s t-tests. One-way ANOVA was performed to compare differences between groups. Data represent mean ± SD from ≥3 biological replicates. ESCC, esophageal squamous cell carcinoma; GEO, Gene Expression Omnibus; IHC, immunohistochemistry; RT-qPCR, real-time quantitative PCR; TCGA, The Cancer Genome Atlas; WTAP, Wilms’ tumor 1-associated protein.

To identify the target m^6^A methyltransferase, we conducted preliminary experiments and identified WTAP as a candidate. WTAP expression was significantly elevated in all ESCC cell lines compared to normal esophageal epithelial (Figs. 1*E* and S1). To investigate the potential role of WTAP in ESCC, we first analyzed RNA sequencing data from the The Cancer Genome Atlas (TCGA) and Gene Expression Omnibus (GEO) databases. The results revealed that WTAP expression was significantly elevated in human ESCC tissues compared to normal tissues (Fig. 1*A*). Subsequently, we assessed WTAP protein expression levels in 71 ESCC tissue samples using immunohistochemistry (IHC). The findings demonstrated that WTAP was markedly upregulated in ESCC tissues relative to paired normal tissues (Fig. 1*B*). Clinical data analysis revealed that WTAP expression was significantly higher in poorly differentiated ESCC tissues compared to moderately and well-differentiated tissues (Fig. 1*C*). However, WTAP expression showed no significant association with the clinical stage, tumor, node, metastasis (TNM) stage, age, or sex of the patients (Fig. S2, *A* and *B*). Kaplan–Meier survival analysis further indicated that patients with high WTAP expression exhibited significantly worse overall survival compared to those with low WTAP expression (Fig. 1*D*). Similarly, we assessed WTAP mRNA and protein levels in the normal esophageal epithelial cell line (HET-1A) and ESCC cell lines (KYSE-30, KYSE-410, KYSE-150, KYSE-510, and TE-1) using real-time quantitative PCR (RT-qPCR) and western blot. The results demonstrated that WTAP expression was significantly elevated in all ESCC cell lines compared to the normal esophageal epithelial cell line (Fig. 1, *E* and *F*). Based on the higher expression levels of WTAP, KYSE-150, and KYSE-510 cell lines were selected for subsequent experiments. In summary, WTAP is highly expressed in both ESCC tissues and cells and is associated with poor prognosis, suggesting that WTAP may serve as a potential oncogenic factor and prognostic biomarker in ESCC.

### WTAP exerts oncogenic effects both *in vivo* and *in vitro*

**Figure 2:**
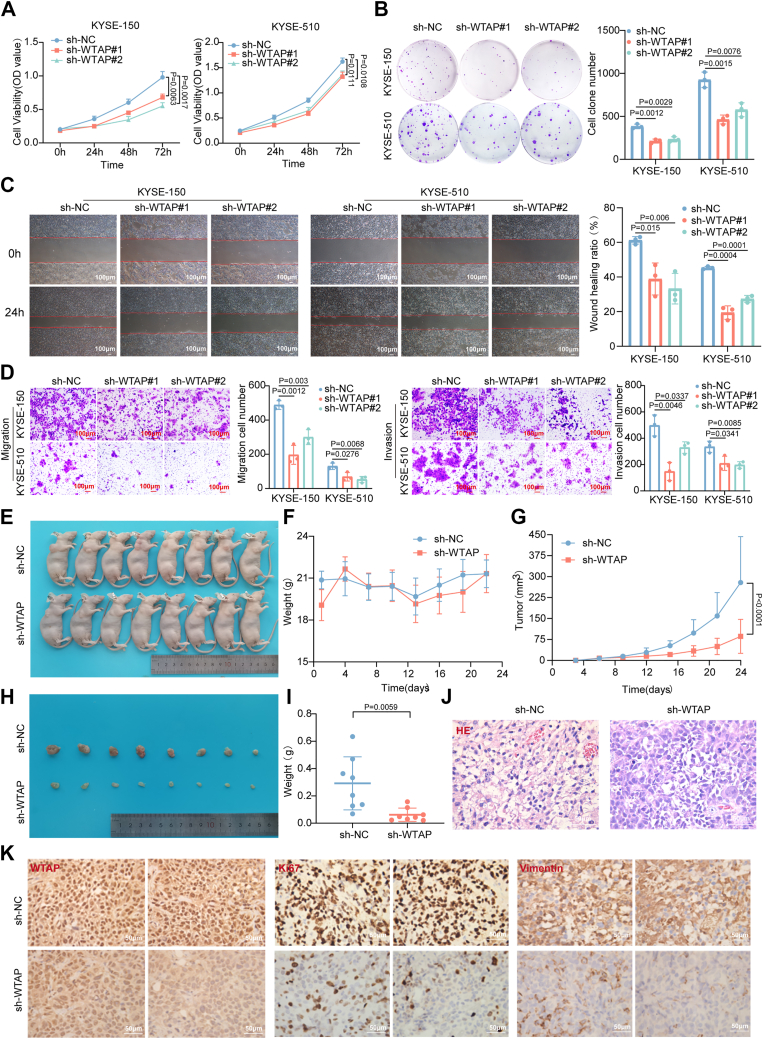
**WTAP exerts oncogenic effects both *in vivo* and *in vitro*.***A*, CCK-8 assay to assess the impact of WTAP knockdown on cell viability in ESCC cell lines (n = 3), *p* < 0.05. *B*, colony formation assay to evaluate the effect of WTAP knockdown on the proliferative capacity of ESCC cell lines (n = 3), *p* < 0.05. *C* and *D*, scratch wound healing and Transwell assays to examine the effect of WTAP knockdown on the migratory and invasive abilities of ESCC cells (n = 3), *p* < 0.05. *E*, schematic diagram of nude mice in the sh-NC and sh-WTAP groups (n = 8 in every group). *F*, comparison of body weight of nude mice between the sh-NC and sh-WTAP groups. *G*, tumor growth curve analysis of nude mice from the sh-NC and sh-WTAP groups, *p* < 0.0001. *H*, schematic of subcutaneous tumors in nude mice from the sh-NC and sh-WTAP groups. *I*, comparison of tumor weight and body weight of nude mice between the sh-NC and sh-WTAP groups, *p* < 0.01. *J*, H&E staining of tumors from the sh-NC and sh-WTAP groups. *K*, IHC analysis of WTAP, Ki-67, and vimentin expression in tumors from the sh-NC and sh-WTAP groups. The differences between the two groups were investigated using Student’s t-tests. One-way ANOVA was performed to compare differences between groups. Data represent mean ± SD from ≥3 biological replicates. CCK-8, Cell Counting Kit-8; ESCC, esophageal squamous cell carcinoma; IHC, immunohistochemistry; WTAP, Wilms’ tumor 1-associated protein.

Given the high expression of WTAP in ESCC tissues and cells, we hypothesize that WTAP may play an oncogenic role in ESCC. To test this, we established WTAP knockdown models in KYSE-150 and KYSE-510 cells using shRNA. Transfection efficiency was verified by RT-qPCR and western blot (Fig. S3, *A* and *B*). Subsequently, cell viability and proliferation were assessed using Cell Counting Kit-8 (CCK-8) and colony formation assays in KYSE-150 and KYSE-510 cells (Fig. 2, *A* and *B*). As expected, the knockdown of WTAP significantly inhibited cell viability and proliferation. Furthermore, scratch wound healing and Transwell assays demonstrated that the downregulation of WTAP markedly suppressed the migratory and invasive abilities of ESCC cells (Fig. 2, *C* and *D*). To further investigate the biological function of WTAP *in vivo*, we established stable KYSE-150 cell lines infected with either sh-NC or sh-WTAP. These cells were then subcutaneously injected into the flanks of nude mice. After 24 days of observation, the mice were euthanized (Fig. 2*E*). We found no significant difference in body weight between the two groups (Fig. 2*F*). In addition, both tumor volume and weight were significantly reduced in the sh-WTAP group compared to the sh-NC group (Fig. 2, *G*–*I*). H&E staining confirmed the successful establishment of the ESCC model in nude mice, with the sh-WTAP group showing a significant reduction in tumor necrosis compared to the sh-NC group (Fig. 2*J*). IHC staining demonstrated that, compared to the NC group, the sh-WTAP group showed downregulation of WTAP, along with a marked reduction in the expression of tumor markers Ki-67 and vimentin (Figs. 2*K* and S3*C*). Collectively, these results demonstrate that WTAP promotes ESCC tumor growth and metastasis both *in vitro* and *in vivo*, highlighting its oncogenic potential.

### WTAP mediates the m^6^A modification of circRNA_404908 and affects its expression and stability

**Figure 3:**
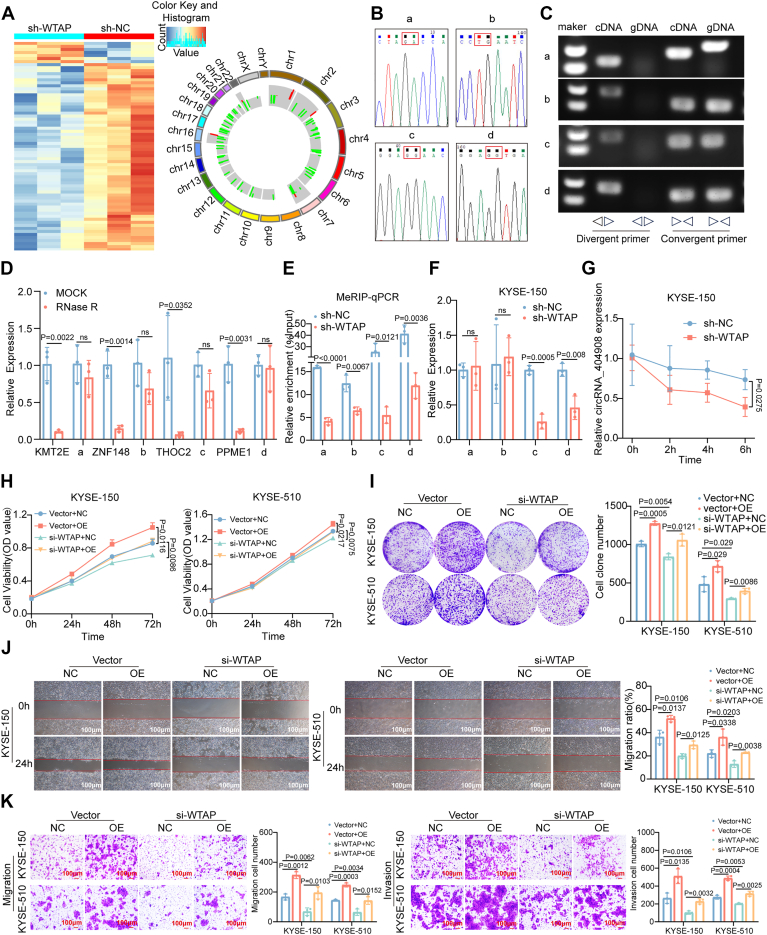
**WTAP mediates the m^6^A modification of circRNA_404908 and affects its expression and stability.***A*, heatmap and Circos plot analysis of differential circRNAs in m^6^A levels using human m^6^A-circRNA epitranscriptomic microarray. *B*, Sanger sequencing analysis of the four exonic circRNAs with altered m^6^A levels. *C*, validation of the circular characteristics of the four exonic circRNAs by agarose gel electrophoresis. *D*, RNase R assay to verify the circular structure of the four exonic circRNAs (n = 3), *p* < 0.05. *E*, MeRIP-qPCR analysis of changes in m^6^A levels of circRNAs following WTAP knockdown (n = 3), *p* < 0.05. *F*, RT-qPCR analysis of expression level changes in circRNAs after WTAP knockdown (n = 3), *p* < 0.01. *G*, the actinomycin D experiment was used to detect the stability changes of circRNA_404908 after the knockdown of WTAP (n = 3), *p* < 0.05. *H* and *I*, plate cloning and CCK-8 assays to assess the effect of circRNA_4040908 overexpression and/or si-WTAP on cell viability and proliferation in KYSE-150 and KYSE-510 cells (n = 3), *p* < 0.05. *J* and *K*, cell scratch and Transwell assays were performed to evaluate the effects of circRNA_4040908 overexpression and/or si-WTAP on migration and invasion in KYSE-150 and KYSE-510 cells (n = 3), *p* < 0.05. The labels a, b, c, and d correspond to circRNA_104455, circRNA_007557, circRNA_105029, and circRNA_4040908, respectively. The differences between the two groups were investigated using Student’s t-tests. One-way ANOVA was performed to compare differences between groups. Data represent mean ± SD from ≥3 biological replicates. CCK-8, Cell Counting Kit-8; m^6^A, N6-methyladenosine; MeRIP-qPCR, methylated RNA immunoprecipitation followed by quantitative PCR; RT-qPCR, real-time quantitative PCR; WTAP, Wilms’ tumor 1-associated protein.

WTAP is a key component of the m^6^A methyltransferase complex. To elucidate the potential mechanisms by which WTAP contributes to ESCC progression, we performed human m^6^A-circRNA epitranscriptomic microarray analysis to investigate changes in m^6^A modifications following WTAP knockdown in KYSE-150 cells. Microarray data revealed that, compared to the control group, 62 circRNAs showed decreased m^6^A modifications, whereas seven circRNAs exhibited increased m^6^A levels in the sh-WTAP group (|FC| ≥ 1.2, *p* < 0.05) (Fig. 3*A*). We selected the top five circRNAs with the most significant downregulation of m^6^A levels for circularization validation, including hsa_circRNA_104455, hsa_circRNA_000454, hsa_circRNA_007557, hsa_circRNA_105029, and hsa_circRNA_4040908. Notably, hsa_circRNA_000454, identified as the only intron-derived circRNA, was excluded from further analysis. We confirmed the circular characteristics of four exonic circRNAs (circRNA_104455, circRNA_007557, circRNA_105029, and circRNA_4040908) through Sanger sequencing, agarose gel electrophoresis, and RNase R digestion assays. These analyses validated that all four circRNAs adopt a circRNA structure (Fig. 3, *B*–*D*). Subsequently, methylated RNA immunoprecipitation followed by quantitative PCR (MeRIP-qPCR) further validated that knockdown of WTAP in KYSE-150 cells led to a significant reduction in m^6^A modification levels of circRNA_104455, circRNA_007557, circRNA_105029, and circRNA_4040908. In addition, RT-qPCR analysis revealed that the expression levels of circRNA_105029 and circRNA_4040908 were significantly decreased following WTAP knockdown (Fig. 3, *E* and *F*). Subsequent preliminary results from a series of cell functional assays showed that circRNA_105029 did not exhibit significant biological functions in the ESCC cell lines (Fig. S4). In contrast, circRNA_4040908 demonstrated a cancer-promoting role, consistent with our expectations, in the ESCC cell lines (Figs. S5 and S6). Meanwhile, to further investigate the relationship of WTAP in regulating the expression level of circRNA_404908, we discovered through the actinomycin D experiment that after the knockdown of WTAP, the stability of circRNA_404908 declined. WTAP influences its expression by affecting the stability of circRNA_404908 (Fig. 3*G*).

According to the circRNA annotation from Circle Primer, hsa_circRNA_4040908 is derived from exons 5 to 9 of the parental gene PPME1 (Fig. S5*A*). Compared to the linear PPME1 transcript, circRNA_4040908 demonstrated greater stability after RNase R exonuclease treatment (Fig. 3*D*). We assessed the transfection efficiency of si-circRNA_404908 and circRNA_404908 overexpression lentivirus in KYSE-150 and KYSE-510 cells using RT-qPCR (Fig. S5*B*). Functional assays revealed that the knockdown of circRNA_404908 reduced cell viability, proliferation, and migration/invasion capabilities in ESCC cells, while overexpression of circRNA_404908 enhanced these functions and counteracted the effects of si-WTAP on cell viability, proliferation, and migration/invasion (Figs. 3, *H*–*K*, S5, *C* and *D*, and S6). These findings suggest that WTAP mediates the m^6^A modification of circRNA_404908, thereby regulating its expression and stability. These results indicate that circRNA_404908 acts as a key downstream effector of WTAP-mediated m^6^A modification, contributing to the oncogenic role of WTAP in ESCC.

### circRNA_4040908 acts as a sponge for miR-3059-5p in ESCC

**Figure 4:**
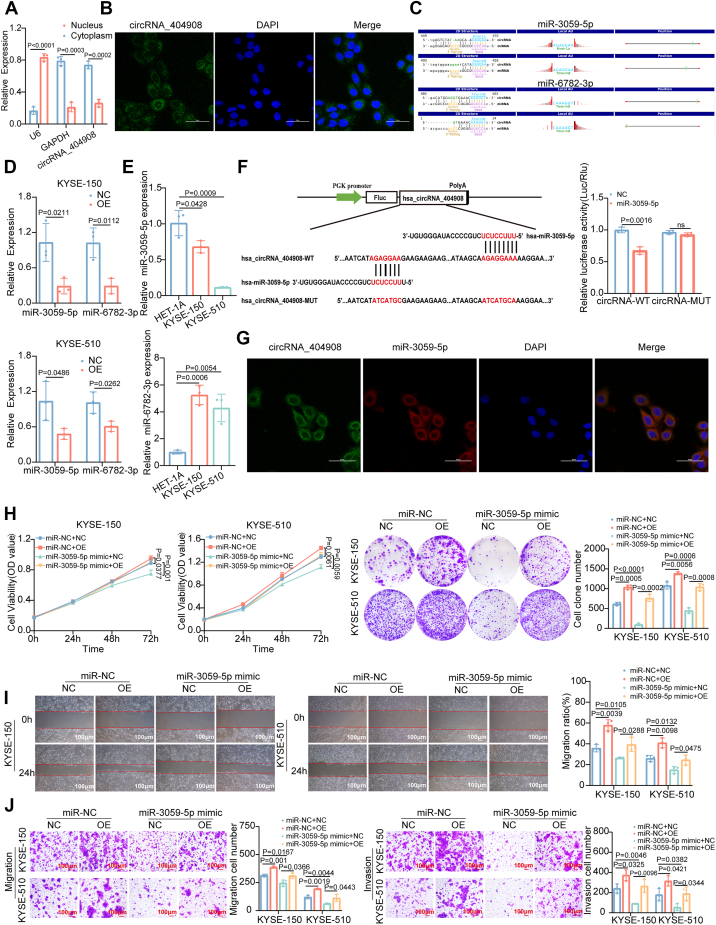
**circRNA_4040908 acts as a sponge for miR-3059-5p in ESCC.***A*, localization of circRNA_4040908 in ESCC cells (n = 3), *p* < 0.05. *B*, FISH analysis confirming the localization of circRNA_4040908 in ESCC cells (n = 3). *C*, schematic diagram showing the interaction of both two miRNAs and circRNA_4040908. *D*, expression changes of the two miRNAs following overexpression of circRNA_4040908 (n = 3), *p* < 0.05. *E*, expression levels of miR-6782-3p and miR-3059-5p in KYSE-150 and KYSE-510 compared to HET-1A (n = 3), *p* < 0.05. *F*, a schematic diagram of the interaction between miR-3059-5p and circRNA_4040908 and the validation of the interaction by the dual-luciferase reporter assay (n = 3), *p* < 0.05. *G*, FISH analysis confirming the colocalization of circRNA_4040908 and miR-3059-5p (n = 3). *H*, colony formation and CCK8 assays evaluating the effects of miR-3059-5p mimic and/or overexpression of circRNA_4040908 on cell viability and proliferation in ESCC cells (n = 3), *p* < 0.05. *I* and *J*, scratch wound healing and Transwell assays assessing the effects of miR-3059-5p mimic and/or overexpression of circRNA_4040908 on migration and invasion in ESCC cells (n = 3), *p* < 0.05. The differences between the two groups were investigated using Student’s t-tests. One-way ANOVA was performed to compare differences between groups. Data represent mean ± SD from ≥3 biological replicates. CCK-8, Cell Counting Kit-8; circRNA, circular RNA; ESCC, esophageal squamous cell carcinoma; miRNA, microRNA.

The potential mechanism of circRNA is generally determined by its localization. Through subcellular fractionation, we found that circRNA_4040908 is predominantly located in the cytoplasm, which was further confirmed by FISH analysis (Fig. 4, *A* and *B*). The competing endogenous RNA (ceRNA) mechanism is commonly observed in cytoplasmic circRNAs. Using the miRNA target prediction software developed by Kangcheng Bio, based on TargetScan and miRanda, we identified two potential miRNAs that may interact with circRNA_4040908 (Fig. 4*C*). RT-qPCR analysis revealed that, compared to the control group, overexpression of circRNA_4040908 significantly reduced the expression levels of miR-3059-5p and miR-6782-3p. Furthermore, circRNA_4040908 exhibited a negative correlation with both of the two miRNAs (Fig. 4*D*). RT-qPCR analysis showed that, compared to the normal esophageal epithelial cell line HET-1A, miR-6782-3p was highly expressed in ESCC cell lines, while miR-3059-5p was lowly expressed in these cell lines (Fig. 4*E*). The only miRNA that aligned with the expectations of this study, miR-3059-5p, was further analyzed for its binding sites. Dual-luciferase reporter assays confirmed that miR-3059-5p interacts with circRNA_4040908 and shows a negative correlation (Fig. 4*F*). The FISH analysis demonstrated that miR-3059-5p colocalizes with circRNA_4040908 in the cytoplasm (Fig. 4*G*). CCK8 and colony formation assays showed that miR-3059-5p mimic inhibits cell viability and proliferation in ESCC cells. Transwell and cell scratch assays further demonstrated that miR-3059-5p mimic suppresses migration and invasion of ESCC cells, whereas the miR-3059-5p inhibitor exerted the opposite effects (Fig. S7). The experiments showed that miR-3059-5p mimic can counteract the promoting effects of circRNA_4040908 on cell viability, proliferation, migration, and invasion in ESCC cells (Fig. 4, *H*–*J*). Further examination of circRNA_404908 and miR-3059-5p expression in ESCC revealed that circRNA_404908 was highly expressed in ESCC tissues, whereas miR-3059-5p was relatively low-expressed, which was consistent with the expectation of this study (Fig. S8). Together, these results suggest that circRNA_4040908 functions as a ceRNA by sponging miR-3059-5p, thereby promoting ESCC progression.

### circRNA_4040908 promotes ESCC progression by regulating the miR-3059-5p/ANO1 axis

**Figure 5:**
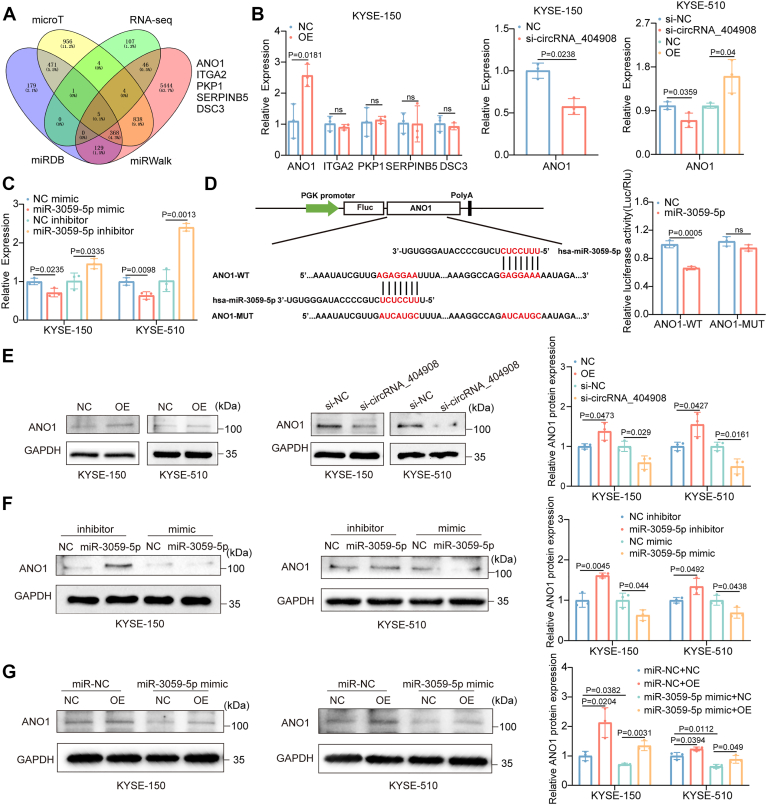
**ANO1 is a common target of circRNA_404908/miR-3059-5p.***A*, screening of downstream target genes of miR-3059-5p using microT, miRDB, and miRWalk databases combined with RNA-seq analysis. *B*, changes in the mRNA levels of ANO1 following circRNA_404908 overexpression in KYSE-150 and KYSE-510 cells (n = 3), *p* < 0.05. *C*, RT-qPCR analysis of the relationship between miR-3059-5p mimic, miR-3059-5p inhibitor, and ANO1 expression levels (n = 3), *p* < 0.05. *D*, schematic of the binding sites of miR-3059-5p with ANO1 and validation by dual-luciferase reporter assay (n = 3), *p* < 0.001. *E*, changes in ANO1 protein levels following circRNA_404908 overexpression and knockdown (n = 3), *p* < 0.05. *F*, western blot analysis to assess the effects of miR-3059-5p mimic and miR-3059-5p inhibitor on ANO1 protein expression (n = 3), *p* < 0.05. *G*, western blot analysis to evaluate the effects of miR-3059-5p mimic and/or circRNA_404908 overexpression on ANO1 protein levels (n = 3), *p* < 0.05. The differences between the two groups were investigated using Student’s t-tests. One-way ANOVA was performed to compare differences between groups. Data represent mean ± SD from ≥3 biological replicates. circRNA, circular RNA; RT-qPCR, real-time quantitative PCR.

To identify the downstream target genes of miR-3059-5p, we performed a comprehensive analysis using the microT, miRDB, and miRWalk databases, along with our previous RNA-seq data on ESCC (GSE263647), which revealed five potential target genes: ANO1, ITGA2, PKP1, SERPINB5, and DSC3 (Fig. 5*A*). To further screen and validate the potential target genes, we measured the mRNA expression levels of the five candidate genes following overexpression of circRNA_4040908 using RT-qPCR. The results showed that only the expression of ANO1 was significantly upregulated, while the other four genes did not show any significant changes. Similarly, silencing circRNA_4040908 led to a significant decrease in the mRNA levels of ANO1 (Fig. 5*B*). The expression level of miR-3059-5p was negatively correlated with ANO1 (Fig. 5*C*). Prediction of the binding sites between miR-3059-5p and ANO1, followed by mutation analysis, was conducted using a dual-luciferase reporter assay. The results confirmed the interaction between miR-3059-5p and ANO1 (Fig. 5*D*). Western blot analysis further confirmed that ANO1 is a common downstream target of circRNA_404908/miR-3059-5p. The protein levels of ANO1 were regulated by both circRNA_404908 and miR-3059-5p. Rescue experiments showed that miR-3059-5p mimic could inhibit the upregulation of ANO1 induced by circRNA_404908 overexpression (Fig. 5, *E*–*G*). These findings indicate that circRNA_4040908 promotes ESCC progression by modulating the miR-3059-5p/ANO1 axis, highlighting a critical regulatory pathway in tumor development.

### ANO1 is highly expressed in ESCC and is associated with poor prognosis

**Figure 6:**
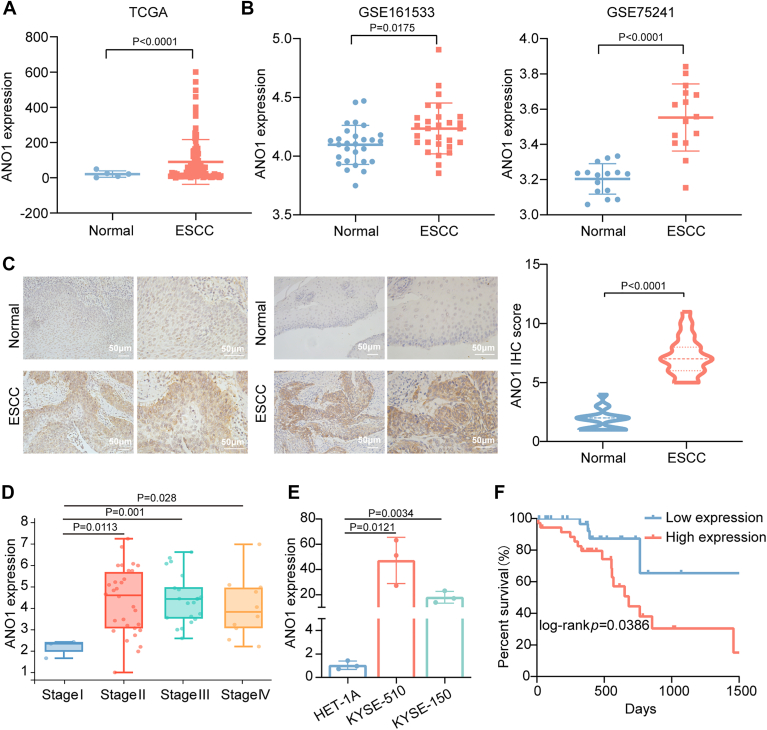
**High expression of ANO1 in ESCC correlates with poor prognosis.***A* and *B*, analysis of ANO1 expression in ESCC using TCGA and GEO Databases (GSE161533 and GSE75241). *C*, IHC analysis of ANO1 expression in normal and ESCC (n = 44), *p* < 0.0001. *D*, relationship between ANO1 expression and clinical staging in TCGA Database, *p* < 0.05. *E*, tissues expression of ANO1 in HET-1A and ESCC cell lines (KYSE-150 and KYSE-510) (n = 3), *p* < 0.05. *F*, Kaplan-Meier survival analysis of ANO1 expression and prognosis in ESCC patients, *p* < 0.05. The differences between the two groups were investigated using Student’s t-tests. One-way ANOVA was performed to compare differences between groups. Data represent mean ± SD from ≥3 biological replicates. ESCC, esophageal squamous cell carcinoma; IHC, immunohistochemistry; GEO, Gene Expression Omnibus; TCGA, The Cancer Genome Atlas.

Analysis of data from the TCGA and GEO databases revealed a significant increase in the expression level of ANO1 in ESCC compared to normal tissues (Fig. 6, *A* and *B*). IHC analysis of 44 paired ESCC patient tissues revealed that, compared to normal tissues, ANO1 expression was significantly increased in ESCC tissues (Fig. 6*C*). In addition, analysis of clinical data from TCGA revealed that ANO1 expression was significantly higher in stage II, stage III, and stage IV compared to stage I (Fig. 6*D*). However, ANO1 expression showed no significant association with the degree of differentiation, TNM stage, age, or sex of the patients (Fig. S9). RT-qPCR analysis showed that the expression level of ANO1 was significantly higher in the ESCC cell lines KYSE-150 and KYSE-510 compared to HET-1A (Fig. 6*E*). Kaplan–Meier analysis showed that patients with high ANO1 expression had a poorer overall survival compared to those with low ANO1 expression (Fig. 6*F*). Together, these results suggest that ANO1 is upregulated in ESCC and may serve as a potential prognostic biomarker and therapeutic target.

## Discussion

EC is one of the most prevalent malignancies of the digestive system globally, with both high incidence and mortality rates. The disease is often asymptomatic in its early stages, resulting in late-stage diagnosis and poor 5-year survival outcomes (21). Surgical resection, chemotherapy, and radiotherapy are the main treatment modalities for ESCC and are widely used in clinical practice. Despite advances in diagnostic techniques and treatment strategies in recent years, and several clinical breakthroughs in ESCC management, significant challenges remain in improving patient outcomes (22, 23).

Since the 1950s, various RNA methylation modifications, including N6-methyladenosine (m^6^A), 5-methylcytosine (m^5^C), N7-methylguanosine (m^7^G), N1-methyladenosine (m^1^A), and 2′-O-methylation, have been identified and characterized (24, 25). Among these, m^6^A is one of the most abundant and biologically significant RNA modifications in eukaryotes. It is introduced by methylation at the N6 position of adenosine in various RNA species, including miRNA, mRNA, tRNA, rRNA, long noncoding RNA (lncRNA), and circRNA (26). The m^6^A modification is a dynamic and reversible process, mediated by the deposition of m^6^A methyltransferases (writers) such as METTL3, METTL14, and WTAP, the removal by m^6^A demethylases (erasers) like fat-mass and obesity-associated protein (FTO) and alkB homolog 5 (ALKBH5), and the recognition by reader proteins, including YTH domain-containing proteins (27). WTAP primarily assists METTL3 and METTL14 in targeting nuclear speckles, while also harboring independent methylation sites that enable the specific methylation of certain m^6^A residues (28). Several studies have highlighted the pivotal role of WTAP-mediated m^6^A modifications in regulating tumor proliferation, invasion, and apoptosis. For example, PRMT1 modulates NDUFS6 expression in an m^6^A-dependent manner through its interaction with WTAP, thereby promoting MM cell proliferation and oxygen consumption rate levels, while simultaneously reducing apoptosis and reactive oxygen species levels (29). Furthermore, WTAP diminishes oxaliplatin chemotherapy sensitivity in colorectal cancer by preventing PANoptosis (30). WTAP regulates autophagy in colon cancer cells by inhibiting FLNA through m^6^A modification (31). Moreover, WTAP-mediated m^6^A modification of ULK1 enhances its mRNA stability in an IGF2BP3-dependent manner, resulting in increased ULK1 expression and augmented mitochondrial autophagy in epithelial ovarian cancer (32).

Although the role of WTAP has been extensively studied, its mechanistic involvement in ESCC remains poorly understood. In this study, we investigate the role of WTAP and the potential mechanisms by which WTAP-mediated m^6^A modifications contribute to the progression and metastasis of ESCC.

Initially, we performed RT-qPCR screening in preliminary experiments to assess the expression levels of m^6^A writers in ESCC cell lines. Among these, WTAP was the only writer consistently exhibiting significantly higher expression across all ESCC cell lines compared to HET-1A cells. Analysis of TCGA and GEO databases, along with IHC, revealed that WTAP is overexpressed in ESCC tissues and is associated with poor prognosis. Consistent with this, western blotting and RT-qPCR experiments confirmed the elevated expression of WTAP in ESCC cell lines. Functional studies further demonstrated that WTAP exerts protumor effects both *in vitro* and *in vivo*. Previous research has shown that WTAP promotes the methylation of noncoding RNAs, thereby enhancing their stability (33, 34, 35). Based on these findings, we next investigated the impact of WTAP-mediated m^6^A modifications on the development and progression of ESCC.

Recent advances in research have brought significant attention to circRNAs due to their stable, circular structure (36, 37). circRNAs are aberrantly expressed in various cancers and are implicated in oncogenesis (38, 39, 40). Previous studies have highlighted the crucial role of m^6^A modifications in regulating the expression and function of circRNAs. For example, m^6^A modification of circUGGT2 by METTL14 promotes gastric cancer tumorigenesis and cisplatin (DDP) resistance by sponging miR-186-3p and upregulating MAP3K (41). In addition, m^6^A-mediated biogenesis of circ_0032463 enhances the malignant biological behavior of osteosarcoma cells, partly by regulating the miR-145-5p/GFRA1 axis (42). In this study, we employed m^6^A-circRNA epitranscriptomic microarray to detect changes in m^6^A levels of circRNAs following WTAP knockdown. The results were validated through circRNA cyclization assays, MeRIP-qPCR, and RT-qPCR to further screen for circRNAs whose expression decreased with WTAP reduction. Two candidate circRNAs, circRNA_404908, and circRNA_105029, were identified. However, preliminary experiments indicated that circRNA_105029 did not exhibit significant biological activity, prompting us to focus on circRNA_404908. CircRNA_404908 is derived from exons 5 to 9 of the PPME1 gene. To further determine that WTAP regulates circRNA_404908 rather than its parental gene PPME1, knockdown of WTAP showed no significant change in the expression level of PPME1 by RT-qPCR assays (Fig. S10*A*). Functional assays demonstrated that circRNA_404908 protumor effects in ESCC cell lines. WTAP mediates the m^6^A modification of circRNA_404908, and the expression level and stability of circRNA_404908 are positively correlated with WTAP. Moreover, overexpression of circRNA_404908 can partially rescue the inhibitory effects of WTAP knockdown on ESCC cell proliferation, migration, and invasion.

Given the correlation between the mechanism of circRNAs and their cellular localization, nuclear-cytoplasmic separation, and FISH assays revealed that circRNA_404908 is predominantly localized in the cytoplasm. Since cytoplasmic circRNAs are often involved in the ceRNA mechanism, bioinformatics analysis predicted three potential miRNAs that may interact with circRNA_404908. RT-qPCR confirmed a negative correlation between these miRNAs and circRNA_404908. Based on the evidence suggesting that circRNA_404908 acts as an oncogene, we next focused on validating miRNAs with potential tumor-suppressive roles. miR-6782-3p was found to be upregulated in ESCC cell lines, prompting us to investigate the lowly expressed miR-3059-5p. Dual-luciferase reporter assays confirmed that circRNA_404908 directly interacts with miR-3059-5p in a negative correlation. Similarly, PPME1 expression was not significantly correlated with circRNA_404908 and miR-3059-5p (Fig. S10, *B* and *C*). Functional assays demonstrated that miR-3059-5p acts as a tumor suppressor in ESCC, and the miR-3059-5p mimic was able to partially reverse the effects of circRNA_404908 overexpression on cell proliferation, migration, and invasion.

To identify the downstream target genes of circRNA_404908 and miR-3059-5p, we combined bioinformatics databases with RNA-seq analysis and identified five potential target genes. Further validation by RT-qPCR revealed that only ANO1 is regulated by both circRNA_404908 and miR-3059-5p. ANO1, a member of the transmembrane protein 16 (TMEM16) family, also known as TMEM16A, was initially identified as a Ca^2+^-activated chloride channel and has been reported to be highly expressed in various cancer types (43, 44, 45). Similarly, the present study demonstrated that ANO1 was highly expressed in ESCC tissues and was associated with poor prognosis. Studies have indicated that ANO1 promotes growth and metastasis in ESCC, making it a promising therapeutic target for ESCC (46, 47, 48, 49).

**Figure 7:**
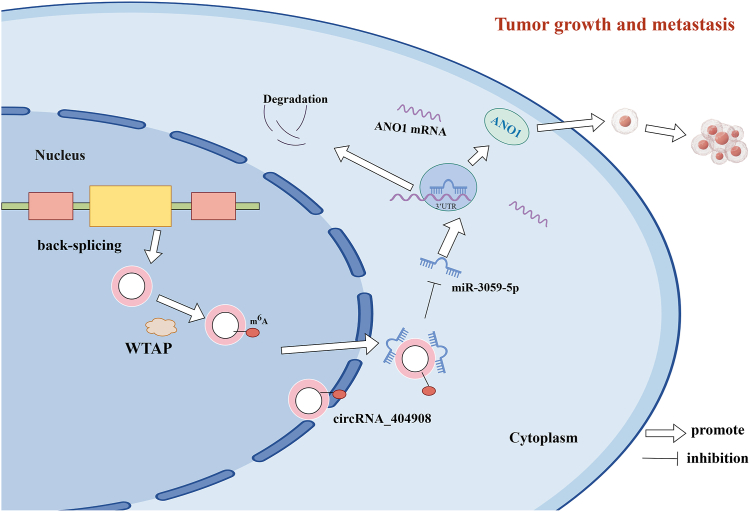
**Schematic representation of the proposed mechanism of WTAP in ESCC.** WTAP increases the m^6^A level of circRNA_404908 and enhances the expression of circRNA_404908, thereby downregulating miR-3059-5p and upregulating ANO1, which contributes to the progression of ESCC. circRNA, circular RNA; ESCC, esophageal squamous cell carcinoma; m^6^A, N6-methyladenosine; WTAP, Wilms’ tumor 1-associated protein.

In summary, WTAP is highly expressed in ESCC tissues and cells, where it exerts protumor effects both *in vitro* and *in vivo*. WTAP mediates the m^6^A modification of circRNA_404908, promoting its expression and stability. CircRNA_404908 further enhances cell proliferation, migration, and invasion in ESCC through the miR-3059-5p/ANO1 axis (Fig. 7).

## Experimental procedures

### Clinical tissues

Clinical and pathological data were obtained from TCGA database, including 93 ESCC patients and five paired tumor tissue samples. Key clinicopathological characteristics, such as age, gender, clinical stage, pathological stage, TNM classification, and survival status, were also collected. In addition, gene expression profiles of 28 and 15 ESCC tumor tissues and their paired normal tissues were downloaded from the GEO database (GSE161533 and GSE75241). The code for data analysis and processing of TCGA and GEO datasets is provided in the supporting information text.

In a previous study, our research group collected six pairs of ESCC and adjacent nontumor tissues from Nanchong Central Hospital for transcriptome sequencing (GSE263647). All patients had not received any treatment before surgery. This study was approved by the Ethics Committee of Nanchong Central Hospital (Approval No. 2019095).

IHC analysis tissue samples were obtained from 106 patients who underwent tumor resection at Nanchong Central Hospital. All selected patients had not received radiotherapy or chemotherapy before or after surgery. The pathological data of ESCC patients included in the IHC analysis are provided in Tables S1 and S2. This study was approved by the Ethics Committee (NSMC Ethical Approval No. 2021–42).

### Cell lines and culture conditions

The human ESCC cell lines KYSE150 and TE-1 were purchased from Shanghai Chinese Academy of Sciences Cell Bank, KYSE30 and KYSE410 were purchased from Hunan Fenghui Biotechnology Co, Ltd, KYSE510 was purchased from Wuhan Shangen Biotechnology Co. Ltd. The ESCC cell lines were cultured in RPMI-1640 medium (Gibco) with 10% fetal bovine serum (Bovogen). Immortalized human normal esophageal epithelial cells HET-1A were cultured in Bronchial Epithelial Cell Growth Medium (Gibco). All cells were cultured at 37 °C and 5% CO_2_ and were regularly tested for *mycoplasma* contamination using the MycoBlue *Mycoplasma* Detector (Vazyme). All cells were validated by short tandem repeat analysis.

### RNA extraction and RT-qPCR

Total RNA was extracted from each group using the FastPure Cell/Tissue Total RNA Isolation Kit (Vazyme). RNA concentration and purity were measured using a spectrophotometer. Subsequently, RNA was reverse-transcribed into complementary DNA using the HiScript III RT SuperMix (Vazyme). RT-qPCR was performed using a two-step method under the following cycling conditions: 95 °C for 3 min, followed by 40 cycles of 95 °C for 10 s, and 60 °C for 30 s. Relative expression levels of target genes were quantified using the 2^−ΔΔCt^ method. For mRNA expression analysis, GAPDH served as the endogenous control for human genes, while U6 was used as the internal reference for miRNA expression. In subcellular fractionation assays, GAPDH and U6 were employed as cytoplasmic and nuclear endogenous controls, respectively. Primer sequences for key genes are listed in Table S3.

### Protein extraction and western blot

Cells from each group were collected and lysed using RIPA lysis buffer (Epizyme) to extract total protein. Protein concentrations were quantified using a BCA Protein Assay Kit (Beyotime). Equal amounts of protein samples were separated *via* 10% SDS-PAGE and subsequently transferred onto nitrocellulose membranes. After blocking with 5% skim milk, membranes were incubated with primary antibodies overnight at 4 °C. The following day, membranes were incubated with horseradish peroxidase (HRP)-conjugated secondary antibodies at room temperature for 1 h. After washing with PBST, protein bands were visualized using an enhanced chemiluminescence reagent and imaged with the Bio-Rad Gel Imaging System. Band intensity was quantified using ImageJ software. Antibodies used in this study were obtained from Proteintech: HRP-conjugated Goat Anti-Rabbit IgG (H + L) (SA00001–2, 1:10,000), GAPDH (10494-1-AP, 1:30,000), WTAP (10200-1-AP, 1:1000) and ANO1 (12652-1-AP, 1:500), The final concentrations of each antibody are as follows: WTAP (0.5 μg/ml), ANO1 (0.9 μg/ml), GAPDH (0.02 μg/ml), and HRP-conjugated Goat Anti-Rabbit IgG (H + L) (0.02 μg/ml).

### IHC staining

IHC staining was performed to detect the expression of WTAP and ANO1 in ESCC tissues and corresponding adjacent normal tissues. Tissue sections were placed in an oven at 65 °C for 1 to 2 h for drying. Deparaffinization and gradient rehydration were carried out using a deparaffinization solution and different concentrations of ethanol, respectively. The sections were then incubated in citrate buffer and subjected to high-pressure antigen retrieval. After cooling, the sections were washed with PBS and incubated with a blocking solution to inhibit endogenous peroxidase activity, followed by PBS washing. After overnight blocking, IHC detection was performed according to the two-step method using a commercial kit. Finally, the slides were mounted for observation. The WTAP and ANO1 primary antibodies were diluted at a ratio of 1:150; the final concentrations were 3.33 μg/ml and 3.00 μg/ml, respectively.

The staining results were scored by two pathologists according to the intensity of staining and the positive area, and the scoring rules were as follows: 4 grades according to the intensity of cell staining, with no positive staining, light yellow, brownish yellow, and brownish brown scores of 0, 1, 2, and 3, respectively; and 4 grades according to the percentage of positive cells, with < 25%, 25%-50%, 50%-75%, and > 75% scores of 1, 2, 3, and 4, respectively. 1, 2, 3, and 4 points, respectively, and the final score was obtained by multiplying these two scores.

### Plate cloning assay

After digesting and centrifuging the cells, the cell count was determined. A total of 1500 cells were seeded into each well of a 6-well plate in 2 ml of culture medium. The cells were cultured in a humidified incubator for 7 to 14 days, with medium changes every other day. Once the cell colonies reached approximately 50 cells, the colonies were fixed, stained, photographed, and counted.

### CCK-8 assay

Cells from each group were collected and seeded at 3000 cells per well in a 96-well plate. After cell attachment, four-time points (0 h, 24 h, 48 h, and 72 h) were selected for analysis. At each time point, the original culture medium was removed, and 100 μl of a mixture consisting of complete culture medium and CCK-8 reagent at a 10:1 ratio was added to each well. The plate was then incubated in the dark at 37 °C for 1 h. Finally, the absorbance (*A*) at 450 nm was measured for each well, and statistical analysis was performed.

### Cell scratch assay

Logarithmically growing KYSE-150 and KYSE-510 cells were seeded into 6-well plates. When the cell density reached 70 to 80%, a scratch was made in the monolayer. After washing with PBS, 2 ml of serum-free medium was added to each well. Images were captured at 0 h and 24 h post scratch, and the cell scratch healing rate was calculated.

### Transwell assay

Cells from each group were digested, centrifuged, and washed twice with PBS. The cells were then resuspended in a serum-free medium and counted. A total of 3 × 10^4^ cells in 200 μl of serum-free medium were added to the upper chamber, and 600 μl of medium containing 10% fetal bovine serum was added to the lower chamber. For cell invasion assays, the Matrigel was added to the upper chamber, and prepared by mixing Matrigel with serum-free medium at a 1:4 ratio. A total of 40 μl of the Matrigel mixture was added to each chamber, and the plate was incubated at 37 °C for 30 min to allow the Matrigel to solidify. Afterward, 200 μl of serum-free cell suspension containing 6 × 10^4^ cells was added. After 24 to 48 h, the cells were fixed with 4% paraformaldehyde, stained with crystal violet, and nonmigratory cells were wiped off. Images were captured, and the number of migrated or invaded cells was counted.

### SiRNA, miRNA mimic and inhibitor transfection, and lentivirus infection

Sh-WTAP, si-circRNA_404908, overexpression lentivirus of circRNA_404908, and miR-3059-5p mimics and inhibitors were purchased from GenePharma, and the sequences are shown in Table S4. The negative vector, si-NC, and miR-NC were used as control groups. For lentiviral infection, when the ESCC cell lines in 6-well plates reached approximately 50% confluence, a mixture of lentivirus and infection reagents was added to the culture medium. After 48 to 72 h, puromycin was applied for selection. For transfection with si-circRNA_404908 and miR-3059-5p mimics or inhibitors, when the cells reached about 50% confluence, the respective siRNA or miRNA mimics/inhibitors were mixed with Lipofectamine 2000 (Invitrogen) and added to the cells. After transfection for 6 h in a serum-free medium, the medium was replaced with a complete culture medium.

### Human m^6^A-circRNA epitranscriptomic microarray

Total RNA from each sample was quantified using the NanoDrop ND-1000, and RNA integrity was assessed using the Bioanalyzer 2100 or by Mops electrophoresis. Sample preparation and microarray hybridization were performed according to Arraystar's standard protocols. Differentially m^6^A-methylated circRNAs between the two comparison groups were identified based on fold change and statistical significance (*p*-value) thresholds. Hierarchical clustering was performed to reveal the distinct m^6^A-methylation patterns among the samples.

### MeRIP-qPCR

Cell pellets containing 1 × 10^7^ cells from each group were collected, and total RNA was extracted using the TRIzol method followed by a quality assessment. RNA samples were then fragmented, and m^6^A-modified RNA fragments were enriched through immunoprecipitation and magnetic bead incubation. The RNA fragments bound to the m^6^A antibody were subsequently purified. Quantitative PCR and data analysis were performed according to the manufacturer's instructions.

### Agarose gel electrophoresis

Molds were prepared, and agarose gel was dissolved by heating. After slight cooling, nucleic acid stain was added and mixed thoroughly. The mixture was poured into the mold, allowed to solidify, and the comb was removed. The gel was placed in the electrophoresis tank, and an electrophoresis buffer was added. DNA samples were mixed with loading buffer and then loaded into the gel wells. Electrophoresis was conducted at 100 V for 30 min, and the results were observed using a gel imager.

### RNase R treatment

Total RNA (2 μg/group) from KYSE-150 cells was treated with RNase R and incubated at 37 °C for 30 min, followed by heat inactivation at 70 °C for 10 min. Equal volumes of RNA were then reverse transcribed, and the expression levels of circRNA and its parental gene mRNA were detected by RT-qPCR.

### Actinomycin D treatment

When the cells in the 6-well plates reached approximately 50% confluence, 5 μg/ml actinomycin D was added to treat the cells. Cells were collected at 0 h, 2 h, and 4 h, with corresponding negative controls at each time point. Total RNA was extracted, and RT-qPCR was performed.

### Nuclear and cytoplasmic RNA separation

Cytoplasmic and nuclear RNA were isolated using the Nuclear and Cytoplasmic RNA Extraction Kit (Thermo Fisher Scientific) according to the manufacturer's instructions. GAPDH and U6 were used as reference genes for the cytoplasm and nucleus, respectively. Finally, data were analyzed using the 2−^ΔΔCt^ method.

### Dual-luciferase reporter assay

Plasmids and corresponding sequences were synthesized and designed by GenePharma. Dual-luciferase reporter plasmids and RNA were transfected into cells at 60%-80% confluence using Lipofectamine 2000 (Invitrogen) according to the manufacturer's instructions. After 48 h, dual-luciferase activity was measured using the Dual-Luciferase Reporter Assay System (Promega). Plasmid sequences are listed in supporting information text.

### RNA FISH

FISH analysis was performed to investigate the localization of circRNA_404908 and miR-3059-5p in ESCC cells. The circRNA_404908 and miR-3059-5p probes were synthesized by GenePharma. RNA FISH was conducted according to the RNA FISH SA-Biotin System Kit (GenePharma) instructions, and images were captured using a fluorescence confocal microscope. The sequences of the circRNA_404908 and miR-3059-5p probes are listed in Table S5.

### *In vivo* tumor model

Male nude mice (5 weeks old) were purchased from Beijing Huafukang Biotechnology Co, Ltd. KYSE-150 cells infected with sh-WTAP and sh-NC (1 × 10^6^ cells) were resuspended in 200 μl of sterile PBS and subcutaneously injected into the right flank of the mice. After 4 weeks, the mice were euthanized, and xenograft tumors were collected for H&E staining and IHC analysis. Tumor volume was calculated using the formula: volume = (length × width^2^)/2. All animal experiments were approved by the Ethics Committee of North Sichuan Medical College (NSMC Ethical Approval No. 2024–136).

### Bioinformatics database network

ESCC patient information was downloaded from TCGA and GEO databases and analyzed the expression levels of WTAP and ANO1 in ESCC (http://xena.ucsc.edu/getting-started/; https://www.ncbi.nlm.nih.gov/geo/); and by microT (https://dianalab.e-ce.uth.gr/microt_webserver/#/), miRDB (https://mirdb.org/), miRWalk (http://mirwalk.umm.uni-heidelberg.de/) databases to predict potential downstream target genes of miR-3059-5p; mechanism maps were drawn using Figdraw (https://www.figdraw.com/static/index.html#/).

### Statistical analysis

Statistical analyses were performed using GraphPad Prism 8.0. Data that followed a normal distribution are expressed as mean ± SD. Independent t-tests were used for comparisons between two groups, and ANOVAwas used for comparisons among multiple groups. Survival curves were generated using the Kaplan–Meier method and evaluated with the log-rank test. Statistical significance was defined as *p* < 0.05. All major experiments were independently repeated three times.

### Ethics approval and consent to participate

The study was approved by the Institutional Review Board: The Ethics Committee of Nanchong Central Hospital. (including the name of the ethics committee), and the study was performed in accordance with the ethical standards as laid down in the 1964 Declaration of Helsinki and its later amendments or comparable ethical standards. Informed consent was obtained from all patients to be included in the study. Registry and the Registration No. of the study/trial: Clinical tissue specimens Ethical Approval No: [2019095]; Immunohistochemical section Review [2021] No. 42. All institutional and national guidelines for the care and use of laboratory animals were followed.

## Data availability

The datasets used and/or analyzed during the current study are available from the corresponding author upon reasonable request.

## Supporting information

This article contains supporting information.

## Conflict of interest

The authors declare that they have no conflicts of interest with the contents of this article.
