# Supplementary material for: WTAP-mediated m6A methylation of circRNA_404908 promotes esophageal squamous cell carcinoma progression
Source: J Biol Chem. 2025 Jul 22;301(9):110512. doi: 10.1016/j.jbc.2025.110512 (PMC12391797; doi:10.1016/j.jbc.2025.110512)
Supplement: Supporting information [file mmc1.docx]

Plasmid sequences：

>hsa_circ404908-WT

GACATAGCATGGGTGGTGCTATTGCAGTCCACACAGCATCATCCAACCTGGTACCAAGCCTCTTGGGTCTGTGCATGATTGATGTTGTAGAAGGTACAGCTATGGATGCACTTAATAGCATGCAGAATTTCTTACGGGGTCGTCCTAAAACCTTCAAGTCTCTGGAGAATGCTATTGAATGGAGTGTGAAGAGTGGCCAGATTCGAAATCTGGAGTCTGCCCGTGTCTCAATGGTTGGCCAAGTCAAACAGTGTGAAGGAATTACAAGTCCAGAAGGCTCAAAATCTATAGTGGAAGGAATCATAGAGGAAGAAGAAGAAGATGAGGAAGGAAGTGAGTCTATAAGCAAGAGGAAAAAGGAAGATGACATGGAGGTGAAACAAAGGTCAAGAATCCTGAAGATCTGTCTGCAGAAACAATGGCAAAAGACGTTGGCAATGTGGTTGAAGCCATGTATGGGGACCTTCCTCCTCCAATTATGCTGATTG

>hsa_circ404908-MUT

GACATAGCATGGGTGGTGCTATTGCAGTCCACACAGCATCATCCAACCTGGTACCAAGCCTCTTGGGTCTGTGCATGATTGATGTTGTAGAAGGTACAGCTATGGATGCACTTAATAGCATGCAGAATTTCTTACGGGGTCGTCCTAAAACCTTCAAGTCTCTGGAGAATGCTATTGAATGGAGTGTGAAGAGTGGCCAGATTCGAAATCTGGAGTCTGCCCGTGTCTCAATGGTTGGCCAAGTCAAACAGTGTGAAGGAATTACAAGTCCAGAAGGCTCAAAATCTATAGTGGAAGGAATCATATCATGCGAAGAAGAAGATGAGGAAGGAAGTGAGTCTATAAGCAATCATGCAAAGGAAGATGACATGGAGGTGAAACAAAGGTCAAGAATCCTGAAGATCTGTCTGCAGAAACAATGGCAAAAGACGTTGGCAATGTGGTTGAAGCCATGTATGGGGACCTTCCTCCTCCAATTATGCTGATTG

>ANO1-WT

CAAATATCAATTATATGGTAGATTGAGGATTTTTTTTCTGTAGCTCAAAGGTGGAGGGAGTTTATTAGTTAACCAAATATCGTTGAGAGGAATTTAAAATACTGTTACTACCAAAGATTTTTATTAATAAAGGCTTATATTTTGGTAACACTTCTCTATATTTTTACTCACAGGAATGTCACTGTTGGACAATTATTTTAAAAGTGTATAAAACCAAGTCTCATAAATGATATGAGTGATCTAAATTTGCAGCAATGATACTAAACAACTCTCTGAAATTTCTCAAGCACCAAGAGAAACATCATTTTAGCAAAGGCCAGGAGGAAAAATAGAAATAAATTTGTCTTGAAGATCTCATTGATGTGATGTTACATTCCCTTTAATCTGCCAACTGTGGTCAAAGTTCATAGGTGTCGTACATTTCCAT

>ANO1-MUT

CAAATATCAATTATATGGTAGATTGAGGATTTTTTTTCTGTAGCTCAAAGGTGGAGGGAGTTTATTAGTTAACCAAATATCGTTGATCATGCTTTAAAATACTGTTACTACCAAAGATTTTTATTAATAAAGGCTTATATTTTGGTAACACTTCTCTATATTTTTACTCACAGGAATGTCACTGTTGGACAATTATTTTAAAAGTGTATAAAACCAAGTCTCATAAATGATATGAGTGATCTAAATTTGCAGCAATGATACTAAACAACTCTCTGAAATTTCTCAAGCACCAAGAGAAACATCATTTTAGCAAAGGCCAGATCATGCAATAGAAATAAATTTGTCTTGAAGATCTCATTGATGTGATGTTACATTCCCTTTAATCTGCCAACTGTGGTCAAAGTTCATAGGTGTCGTACATTTCCAT

**TCGA downloads and organizes data codes**：

setwd("E:/TCGA") #

library(jsonlite)

library(tidyverse)

library(dplyr)

meta_data <- fromJSON("metadata.cart.2024-01-31.json")

samples_df <- map2_df(meta_data$associated_entities, seq_along(meta_data$associated_entities),

~ tibble(sample_id = .x[,1], file_name = meta_data$file_name[.y]))

count_file_paths <- list.files('gdc_download_20230613_075345.926514/', pattern = '*.tsv', recursive = TRUE)

file_names_only <- sapply(strsplit(count_file_paths, split='/'), function(x) x[2])

expr_matrix <- data.frame()

for (file_index in seq_along(count_file_paths)) {

full_path <- paste0('gdc_download_20230613_075345.926514/', count_file_paths[file_index])

count_data <- read.delim(full_path, fill = TRUE, header = FALSE, row.names = 1)

colnames(count_data) <- count_data[2,]

count_data <- count_data[-(1:6),]

tpm_data <- count_data[6]

sample_id <- samples_df$sample_id[which(samples_df$file_name == file_names_only[file_index])][[1]]

colnames(tpm_data) <- sample_id

if (nrow(expr_matrix) == 0) {

expr_matrix <- tpm_data

} else {

if (!all(rownames(expr_matrix) == rownames(tpm_data))) {

stop("false")

}

expr_matrix <- cbind(expr_matrix, tpm_data)

}

}

for (file_index in seq_along(count_file_paths)) {

full_path <- paste0('gdc_download_20230613_075345.926514/', count_file_paths[file_index])

count_data <- read.delim(full_path, fill = TRUE, header = FALSE, row.names = 1)

colnames(count_data) <- count_data[2,]

count_data <- count_data[-(1:6),]

tpm_data <- count_data[3]

sample_id <- samples_df$sample_id[which(samples_df$file_name == file_names_only[file_index])][[1]]

colnames(tpm_data) <- sample_id

if (nrow(expr_matrix) == 0) {

expr_matrix <- tpm_data

} else {

if (!all(rownames(expr_matrix) == rownames(tpm_data))) {

stop("false ")

}

expr_matrix <- cbind(expr_matrix, tpm_data)

}

}

for (file_index in seq_along(count_file_paths)) {

full_path <- paste0('gdc_download_20230613_075345.926514/', count_file_paths[file_index])

count_data <- read.delim(full_path, fill = TRUE, header = FALSE, row.names = 1)

colnames(count_data) <- count_data[2,]

count_data <- count_data[-(1:6),]

tpm_data <- count_data[7]

sample_id <- samples_df$sample_id[which(samples_df$file_name == file_names_only[file_index])][[1]]

colnames(tpm_data) <- sample_id

if (nrow(expr_matrix) == 0) {

expr_matrix <- tpm_data

} else {

if (!all(rownames(expr_matrix) == rownames(tpm_data))) {

stop("false ")

}

expr_matrix <- cbind(expr_matrix, tpm_data)

}

}

gene_info <- as.matrix(read.delim(paste0('gdc_download_20230613_075345.926514/', count_file_paths[1]), fill = TRUE, header = FALSE, row.names = 1))

genes <- gene_info[-(1:6), 1]

gene_types <- gene_info[-(1:6), 2]

expr_matrix <- cbind(gene_type = gene_types, gene_symbol = genes, expr_matrix)

Bexpr_matrix <- aggregate(. ~ gene_symbol, data = expr_matrix, max)

expr_matrix <- semi_join(expr_matrix, Bexpr_matrix, by = "gene_symbol")

expr_matrix <- Bexpr_matrix

rownames(expr_matrix) <- expr_matrix[, "gene_symbol"]

expr_matrix <- expr_matrix[, -1]

expr_matrix <- data.frame(ID = rownames(expr_matrix), expr_matrix)

colnames(expr_matrix) <- gsub('[.]', '-', colnames(expr_matrix))

expr_matrix <- subset(x = expr_matrix, gene_type == "protein_coding")

expr_matrix <- expr_matrix[, -1]

expr_matrix <- expr_matrix[, -1]

write.table(expr_matrix, 'TCGAAAAAA_LUSC_TPM.txt', sep="\t", quote=FALSE, row.names = FALSE)

**GEO downloads and organizes data codes**：

setwd("E:/GEO)

library(GEOquery)

library(dplyr)

gset <- getGEO("GSE16088",destdir = "E:/GEO",AnnotGPL = F,getGPL = F)

GSE=exprs(gset[[1]])

GSE=as.data.frame(GSE)

ex <- GSE

qx <- as.numeric(quantile(ex, c(0., 0.25, 0.5, 0.75, 0.99, 1.0), na.rm=T))

LogC <- (qx[5] > 100) ||

(qx[6]-qx[1] > 50 && qx[2] > 0) ||

(qx[2] > 0 && qx[2] < 1 && qx[4] > 1 && qx[4] < 2)

if (LogC) { ex[which(ex <= 0)] <- NaN

GSE <- log2(ex)

print("log2 transform finished")}else{print("log2 transform not needed")}

gpl=data.table::fread("GPL96-57554.txt",header = TRUE,sep = "\t")

gpl = gpl[16:nrow(gpl), ]colnames =as.character(gpl[1, ])

gpl = gpl[-1, ]

colnames(gpl) = colnames

ids=gpl[,c("ID","Gene Symbol")]

ids$`Gene Symbol` = gsub("//.*", "", ids$`Gene Symbol`)

ids=ids[ids$ID %in% rownames(GSE),]

GSE=GSE[ids$ID,]

table(rownames(GSE) == ids$ID)

colnames(ids)=c('ID','GeneSymbol')

GSE <- bind_cols(ids, GSE)

any(duplicated(GSE$GeneSymbol))

GSE <- subset(GSE, !duplicated(GSE$GeneSymbol))

any(duplicated(GSE$GeneSymbol))

GSE = GSE[,-1]

View(GSE)

install.packages()

#GSE = GSE[-515,]

write.table(GSE, file = "GSE16088.txt", sep = "\t", row.names = F)

clinical=pData(gset[[1]])

write.csv(clinical,'clinical_GSE16088.csv',row.names = TRUE)
