# Supplementary material for: WTAP-mediated m6A methylation of circRNA_404908 promotes esophageal squamous cell carcinoma progression
Source: J Biol Chem. 2025 Jul 22;301(9):110512. doi: 10.1016/j.jbc.2025.110512 (PMC12391797; doi:10.1016/j.jbc.2025.110512)
Supplement: Supporting Tables [file mmc2.docx]

Table S1 WTAP clinical information

| Pathology No. | Tumor differentiation | Gender | Age | T | N | M |
| --- | --- | --- | --- | --- | --- | --- |
| 02988 | low | Male | 65 | T1 | N2 | M0 |
| 04741 | low | Female | 53 | T1 | N1 | M0 |
| 05693 | low | Female | 80 | T1 | N0 | M0 |
| 15010 | low | Male | 65 | T2 | N0 | M0 |
| 01233 | moderate | Male | 74 | T2 | N3 | M0 |
| 03789 | moderate | Male | 71 | T3 | N1 | M0 |
| 02519 | moderate | Female | 80 | T2 | N2 | M0 |
| 08705 | moderate | Male | 72 | T3 | N1 | M0 |
| 07927 | moderate | Female | 72 | T2 | N0 | M0 |
| 13537 | moderate | Female | 62 | T2 | N2 | M0 |
| 17811 | moderate | Male | 77 | T2 | N1 | M0 |
| 04936 | moderate | Male | 74 | T2 | N1 | M0 |
| 18445 | moderate | Male | 75 | T3 | N2 | M0 |
| 19174 | moderate | Male | 75 | T3 | N0 | M0 |
| 20058 | moderate | Female | 55 | T3 | N2 | M0 |
| 20607 | moderate | Male | 63 | T2 | N1 | M0 |
| 20671 | moderate | Female | 66 | T2 | N2 | M0 |
| 21503 | moderate | Male | 71 | T3 | N2 | M0 |
| 04833 | moderate | Male | 52 | T3 | N2 | M0 |
| 00302 | moderate | Male | 66 | T2 | N2 | M0 |
| 01860 | moderate | Male | 66 | T1 | N1 | M0 |
| 04262 | moderate | Male | 73 | T3 | N1 | M0 |
| 04753 | moderate | Female | 63 | T3 | N0 | M0 |
| 04945 | moderate | Male | 66 | T3 | N2 | M0 |
| 07451 | moderate | Female | 69 | T2 | N0 | M0 |
| 09676 | moderate | Female | 75 | T3 | N1 | M0 |
| 13443 | moderate | Male | 76 | T3 | N1 | M0 |
| 15348 | moderate | Female | 71 | T3 | N0 | M0 |
| 15839 | moderate | Female | 74 | T1 | N1 | M0 |
| 16029 | moderate | Male | 64 | T1 | N1 | M0 |
| 17773 | moderate | Male | 70 | T2 | N1 | M0 |
| 18054 | moderate | Male | 65 | T3 | N2 | M0 |
| 18362 | moderate | Male | 51 | T3 | N2 | M0 |
| 18658 | moderate | Male | 64 | T2 | N1 | M0 |
| 20002 | moderate | Male | 68 | T2 | N0 | M0 |
| 20130 | moderate | Male | 67 | T1 | N0 | M0 |
| 21106 | moderate | Male | 65 | T2 | N0 | M0 |
| 21233 | moderate | Female | 66 | T1 | N0 | M0 |
| 23511 | moderate | Male | 56 | T1 | N0 | M0 |
| 24062 | moderate | Female | 70 | T1 | N0 | M0 |
| 24225 | moderate | Female | 78 | T1 | N0 | M0 |
| 28885 | moderate | Male | 57 | T1 | N1 | M0 |
| 29490 | moderate | Female | 69 | T1 | N0 | M0 |
| 04718 | high | Female | 67 | T3 | N0 | M0 |
| 15251 | high | Male | 62 | T2 | N0 | M0 |
| 15818 | high | Male | 74 | T2 | N1 | M0 |
| 15987 | high | Male | 66 | T2 | N0 | M0 |
| 17653 | high | Female | 74 | T1 | N1 | M0 |
| 17855 | high | Male | 68 | T1 | N0 | M0 |
| 18550 | high | Male | 68 | T3 | N1 | M0 |
| 19575 | high | Male | 66 | T2 | N1 | M0 |
| 20266 | high | Male | 71 | T1 | N0 | M0 |
| 20279 | high | Male | 70 | T1 | N1 | M0 |
| 20324 | high | Male | 80 | T2 | N0 | M0 |
| 23666 | high | Male | 62 | T3 | N1 | M0 |
| 00611 | high | Male | 69 | T2 | N0 | M0 |
| 02912 | high | Male | 73 | T3 | N2 | M0 |
| 03424 | high | Male | 72 | T2 | N0 | M0 |
| 06311 | high | Female | 55 | T3 | N2 | M0 |
| 07738 | high | Female | 66 | T3 | N0 | M0 |
| 08629 | high | Female | 67 | T3 | N0 | M0 |
| 09222 | high | Male | 56 | T3 | N0 | M0 |
| 11658 | high | Male | 68 | T3 | N1 | M0 |
| 13122 | high | Male | 68 | T3 | N0 | M0 |
| 13935 | high | Male | 79 | T3 | N1 | M0 |
| 14296 | high | Male | 66 | T1 | N0 | M0 |
| 15141 | high | Male | 78 | T1 | N0 | M0 |
| 15252 | high | Male | 63 | T3 | N1 | M0 |
| 15639 | high | Male | 71 | T3 | N0 | M0 |
| 18890 | high | Female | 76 | T2 | N0 | M0 |
| 20749 | high | Male | 79 | T3 | N1 | M0 |

Table S2 ANO1 clinical information

| Pathology No. | Tumor differentiation | Gender | Age | T | N | M |
| --- | --- | --- | --- | --- | --- | --- |
| 00569 | high | Male | 65 | T1 | N0 | M0 |
| 01598 | moderate | Male | 55 | T3 | N1 | M0 |
| 01904 | low | Female | 63 | T2 | N2 | M0 |
| 02027 | moderate | Male | 58 | T2 | N0 | M0 |
| 02040 | high | Male | 67 | T3 | N0 | M0 |
| 02108 | high | Female | 77 | T3 | N0 | M0 |
| 02372 | high | Male | 68 | T3 | N0 | M0 |
| 02429 | moderate | Female | 63 | T2 | N0 | M0 |
| 02877 | high | Male | 66 | T3 | N0 | M0 |
| 02988 | low | Male | 65 | T1 | N0 | M0 |
| 03070 | moderate | Male | 67 | T3 | N2 | M0 |
| 03447 | moderate | Male | 65 | T2 | N3 | M0 |
| 03545 | high | Female | 76 | T3 | N0 | M0 |
| 03854 | low | Male | 69 | T3 | N0 | M0 |
| 04312 | high | Male | 73 | T3 | N0 | M0 |
| 05566 | moderate | Male | 67 | T3 | N1 | M0 |
| 05920 | low | Male | 67 | T2 | N3 | M0 |
| 06085 | low | Male | 80 | T3 | N1 | M0 |
| 06420 | moderate | Female | 74 | T2 | N0 | M0 |
| 07457 | high | Female | 69 | T3 | N0 | M0 |
| 07964 | moderate | Male | 69 | T2 | N0 | M0 |
| 18397 | low | Female | 57 | T3 | N2 | M0 |
| 18774 | low | Male | 58 | T3 | N2 | M0 |
| 08993 | low | Female | 55 | T2 | N2 | M0 |
| 09387 | high | Male | 82 | T1 | N1 | M0 |
| 09607 | moderate | Male | 67 | T3 | N0 | M0 |
| 09779 | moderate | Male | 80 | T3 | N0 | M0 |
| 10112 | high | Male | 78 | T3 | N0 | M0 |
| 11260 | low | Female | 72 | T3 | N0 | M0 |
| 11491 | high | Male | 64 | T3 | N0 | M0 |
| 15348 | moderate | Female | 71 | T3 | N0 | M0 |
| 15608 | moderate | Female | 60 | T3 | N2 | M0 |
| 15987 | high | Male | 66 | T2 | N0 | M0 |
| 16029 | moderate | Male | 64 | T1 | N1 | M0 |
| 16830 | high | Male | 64 | T1 | N0 | M0 |
| 17366 | high | Male | 74 | T3 | N0 | M0 |
| 17811 | low | Male | 77 | T3 | N1 | M0 |
| 18257 | high | Male | 70 | T3 | N0 | M0 |
| 18445 | low | Male | 75 | T3 | N2 | M0 |
| 19174 | low | Male | 75 | T3 | N0 | M0 |
| 20324 | high | Male | 80 | T3 | N0 | M0 |
| 20407 | high | Male | 73 | T3 | N1 | M0 |
| 20527 | high | Male | 66 | T3 | N1 | M0 |
| 20607 | low | Male | 63 | T1 | N1 | M0 |

Table S3 The primer sequence

| Gene | Primer sequence (5' 3') |
| --- | --- |
| GAPDH | F: GGAGTCCACTGGCGTCTTCA |
|  | R: GTCATGAGTCCTTCCACGATACC |
| WTAP | F: CTTCCCAAGAAGGTTCGATTGA  R: TCAGACTCTCTTAGGCCAGTTAC |
| RBM15 | F: ACGACCCGCAACAATGAAG  R: GGAAGTCGAGTCCTCACCAC |
| KIAA1429 | F: AAGTGCCCCTGTTTTCGATAG  R: ACCAGACCATCAGTATTCACCT |
| RBM15B | F: TACACGGAGGCTACCAGTACA  R: GTCGTACAGCCCGTAGTAGTC |
| METTL5 | F: AAGGAACTAGAGAGTCGCCTG  R: GCGGCCTGGTAGGATACTG |
| ZC3H13 | F: GTGCCGTAACTGGCTGAAGA  R: CCTTTACCACGAGGTGAAGGG |
| CBLL1 | F: TCCTTGGGTGGTCTTGATGTT  R: CAGGTTTCGCTTTGTTTGCTT |
| METTL3 | F: AGCCTTCTGAACCAACAGTCC |
|  | R: CCGACCTCGAGAGCGAAAT |
| METTL14 | F: GTTGGAACATGGATAGCCGC |
|  | R: CAATGCTGTCGGCACTTTCA |
| METTL16 | F: TGAATTCTGTCAAGGTCGGAC |
|  | R: GTTATGGGTTTTCTCGGTTTCTC |
| hsa-circRNA_404908 | F: GGAATTACAAGTCCAGAAGGCTC  R: ACATGGCTTCAACCACATTGC |
| PPME1 | F: AGGAGGTCATTCTGCCCTTTC  R: TGCCAACGTCTTTTGCCATTG |
| hsa-circRNA_105029 | F: TGCAAAAGTGGTGCGCATAG  R: AGCTTCCGCAATTTCTCGTT |
| THOC2 | F: CGCAGAGTGGATAAAGAACTGG  R: ACATTAGATGCCTGTTCATGCTT |
| hsa-circRNA_104455 | F: CAGCCTAGACCAGAATCCGT  R: GAGGAGGACGAGCACCATAA |
| KMT2E | F: AAATCCAACAGTTATCCCCACC  G: CGCATAGGGCAAACCAATGTA |
| hsa-circRNA_7557 | F: CCTTGCTGCAGATGAAGTGT  R: CTTATGCCATCCGATTCAGGG |
| ZNF148 | F: CAGGACAATGGTTGTAATGGGT  R: GGTGAGGCATACTTCGATCTTGA |
| hsa-miR-3059-5p | F: CGTTTCCTCTCTGCCCCATA  R: AGTGCAGGGTCCGAGGTATT |
| hsa-cmiR-6782-3p | F: CGCACCTTTGTGTCCCCAT  R: AGTGCAGGGTCCGAGGTATT |
| U6 snRNA | F: CAAATTCGTGAAGCGTTCCA  R: AGTGCAGGGTCCGAGGTATT |
| ANO1 | F: ACTACCACGAGGATGACAAGC  R: TCTCTGCACAGCACGTTCC |
| ITGA2 | F: CCTACAATGTTGGTCTCCCAGA  R: AGTAACCAGTTGCCTTTTGGATT |
| PKP1 | F: TTTGCCGTCGGACCAAAAGAT  R: GAACCTCGATTGGAGTGGCTC |
| SERPINB5 | F: AATTCGGCTTTTGCCGTTGAT  R: TGTCACCTTTAGCACCCACTT |
| DSC3 | F: GACCCTCGTGATCTTCAGTCG  R: TCACTTGACCGGATGAGGTCT |

Table S4 SiRNAs, shRNAs, mimic and inhibitor sequence

| Name | Sequences（5'-3'） |
| --- | --- |
| si-WTAP#1 | Sence: CUAAGAGAGUCUGAAGAAAUU  Anti sence: UUUCUUCAGACUCUCUUAGUU |
| si-WTAP#2 | Sence: GCUUUGGAGGGCAAGUACAUU  Anti sence: UGUACUUGCCCUCCAAAGCUU |
| si-circRNA_404908 | Sence: AUGGAGGUGAAACAAAGGUTT  Anti sence: ACCUUUGUUUCACCUCCAUTT |
| sh-WTAP#1 | GGGCAAGTACACAGATCTTAA |
| sh-WTAP#2 | CTGACAAACGGACCAAGTAAT |
| hsa-circRNA_404908 OE | GTGAAACAAAGGTCAAGAATCCTG |
| hsa-miR-3059-5p mimic | Sence: UUUCCUCUCUGCCCCAUAGGGUGU  Anti sence: ACCCUAUGGGGCAGAGAGGAAAUU |
| hsa-miR-3059-5p inhibitor | ACACCCUAUGGGGCAGAGAGGAAA |
| si-NC | Sence: UUCUCCGAACGUGUCACGUTT  Anti sence: ACGUGACACGUUCGGAGAATT |
| sh-NC | TTCTCCGAACGTGTCACGT |
| NC inhibitor | CAGUACUUUUGUGUAGUACAA |

Table S5 Probe sequences

| Gene name | Probe sequences |
| --- | --- |
| circRNA_404908 probe 1 | TT+TCACCTCCATGTCATCT+TCCTTT |
| circRNA_404908 probe 2 | CTT+TGT+TTCACCTCCATGTCATCTT |
| circRNA_404908 probe 3 | CTTGACCT+TTGTT+TCACCTCCATGT |
| miR-3059-5p Probe | ACACCC+TA+TGGGGCAGAGAGGAAA |
