# Supplementary material for: WTAP-mediated m6A methylation of circRNA_404908 promotes esophageal squamous cell carcinoma progression
Source: J Biol Chem. 2025 Jul 22;301(9):110512. doi: 10.1016/j.jbc.2025.110512 (PMC12391797; doi:10.1016/j.jbc.2025.110512)
Supplement: Supporting Figures [file mmc3.docx]

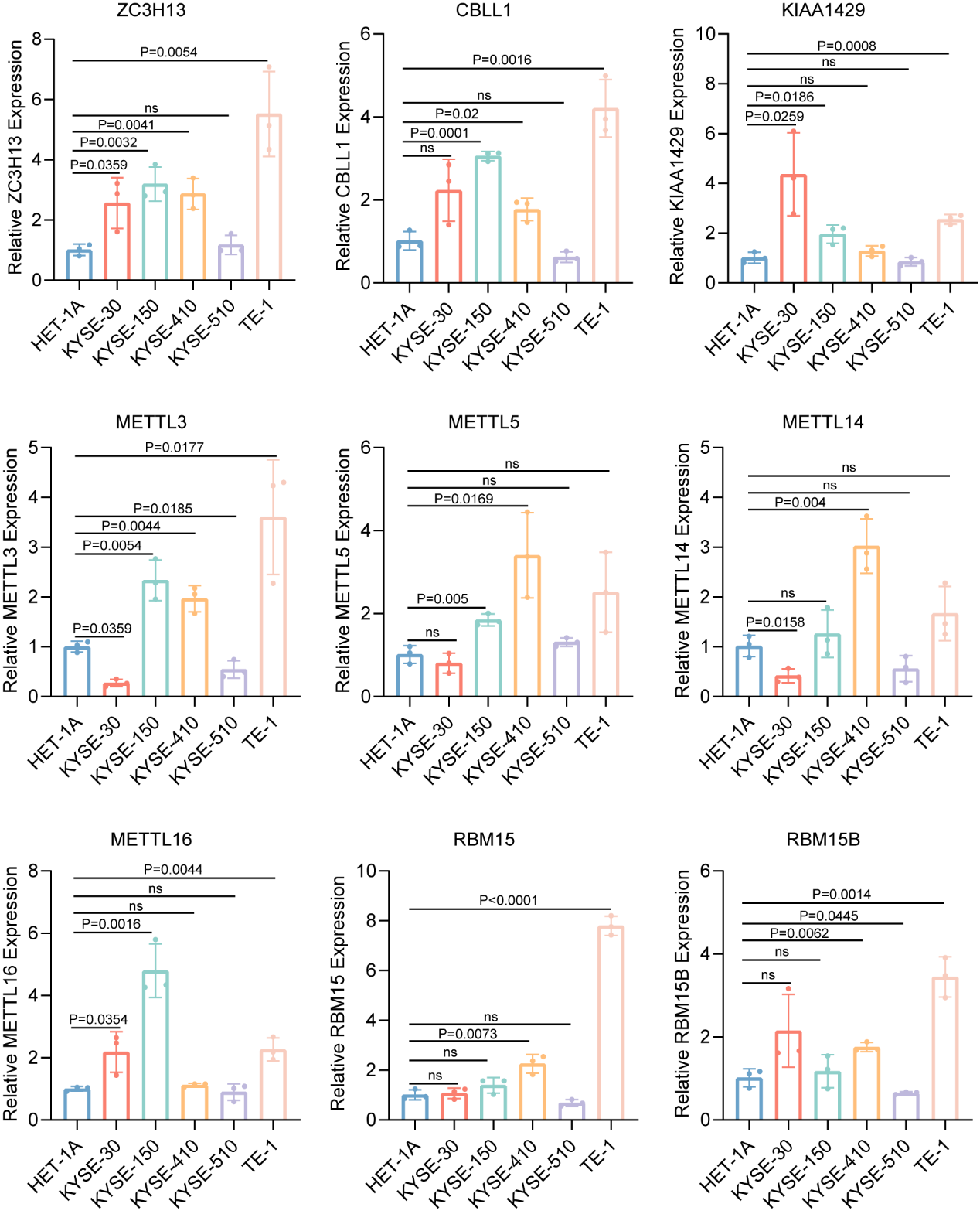


**Fig S1** The expression of m^6^A methyltransferase in ESCC cell lines.

RT-qPCR experiments were used to detect the expression of m^6^A methyltransferase in normal esophageal epithelial cells (HET-1A) and ESCC cell lines.


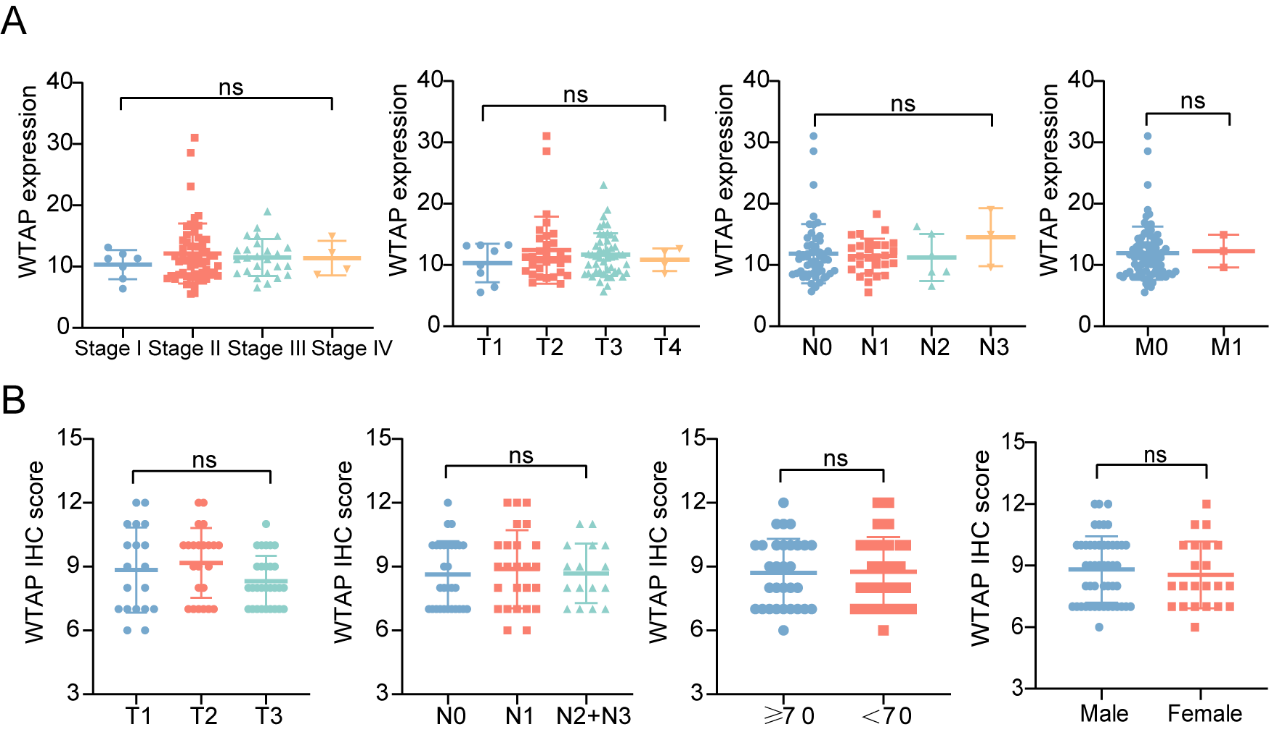


**Fig S2** There is no significant correlation between WTAP expression level and the clinical stage, TNM stage, age and gender of ESCC patients.

**A** Correlation analysis of the expression of WTAP in ESCC patients in the TCGA database with the clinical stage and TNM stage. **B** Analysis of the correlation between the WTAP expression score of ESCC patients in immunohistochemistry and clinical information.


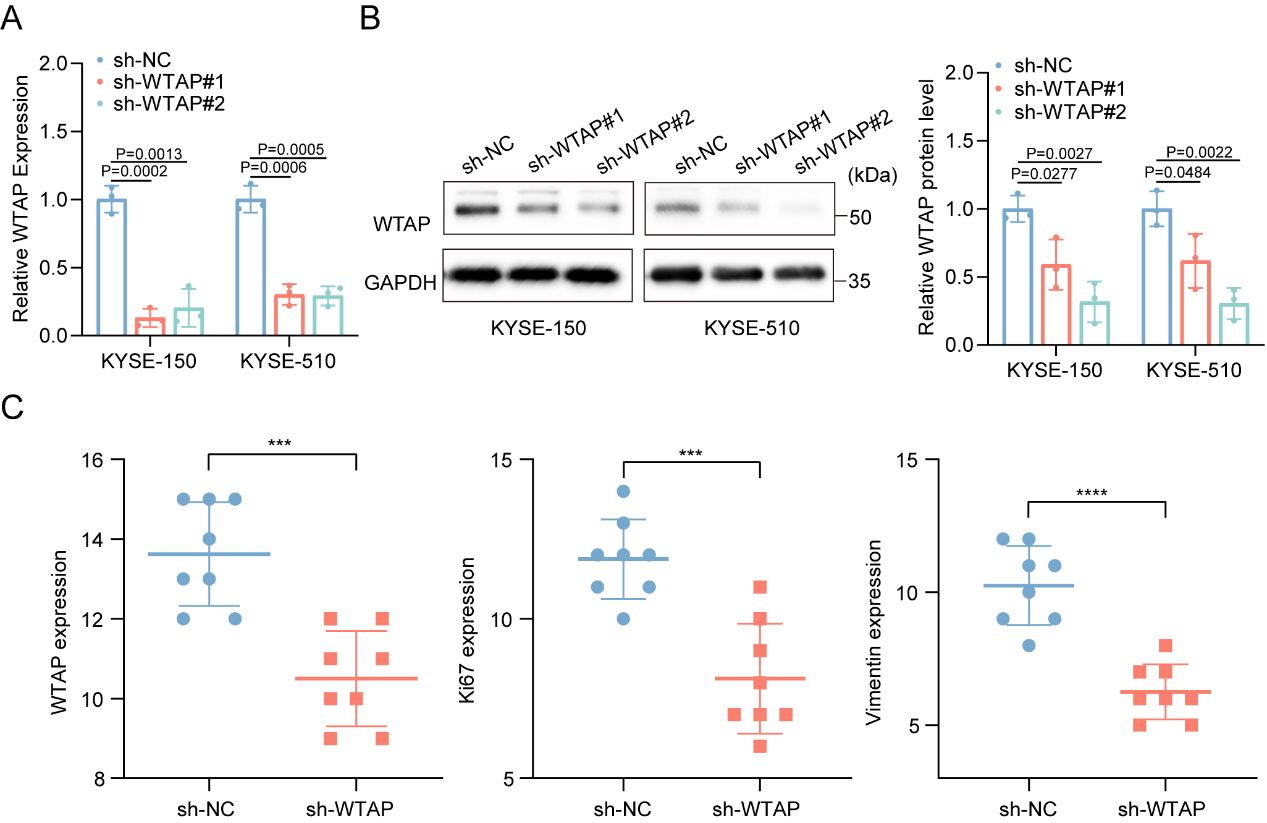


**Fig S3** Verification of the knockdown efficiency of WTAP and the expressions of WTAP, Ki67, and Vimentin in the subcutaneous tumor model tissues of nude mice after knockdown of WTAP were significantly reduced.

**A** RT-qPCR experiment to detect the knockdown efficiency of WTAP at the mRNA level. **B** Western blot experiment to detect the Knockdown efficiency of WTAP at the protein level. **C** Analyze the expression of WTAP and tumor markers Ki67 and Vimentin in the tumor tissues of nude mice in the sh-NC and sh-WTAP groups.


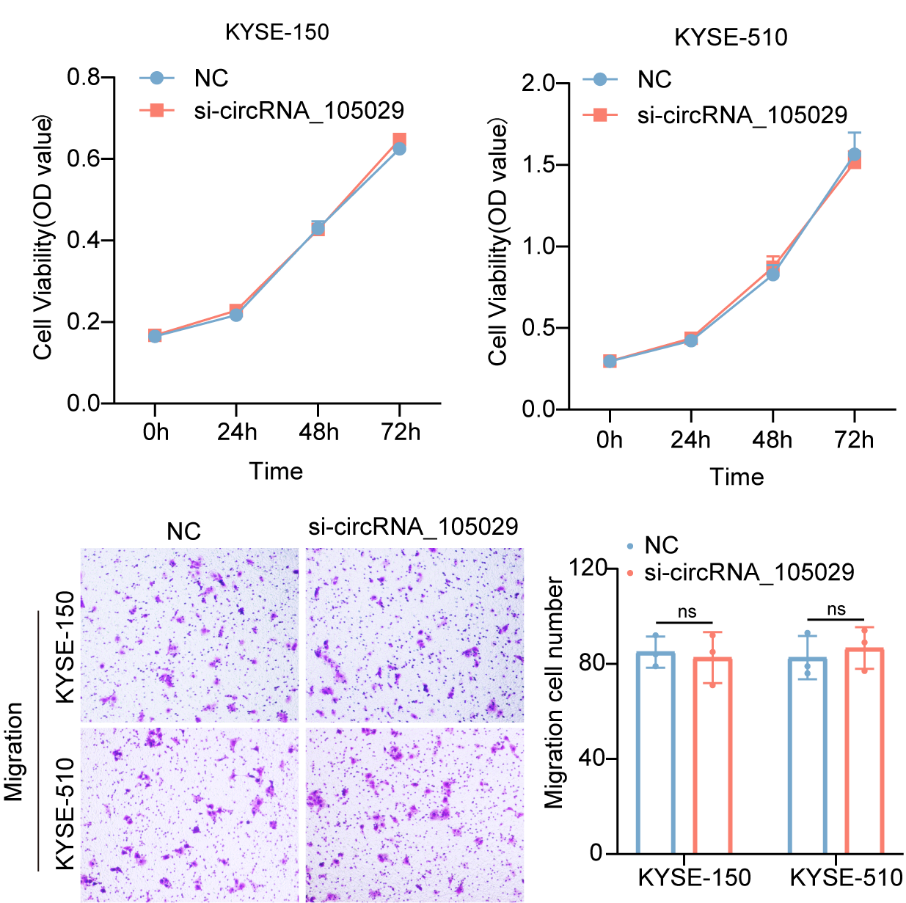


**Fig S4** circRNA_105029 has no obvious biological function in ESCC cells.

CCK-8 and Transwell assays were used to detect the changes in the proliferation activity and migration ability of ESCC cells after knockdown of circRNA_105029.


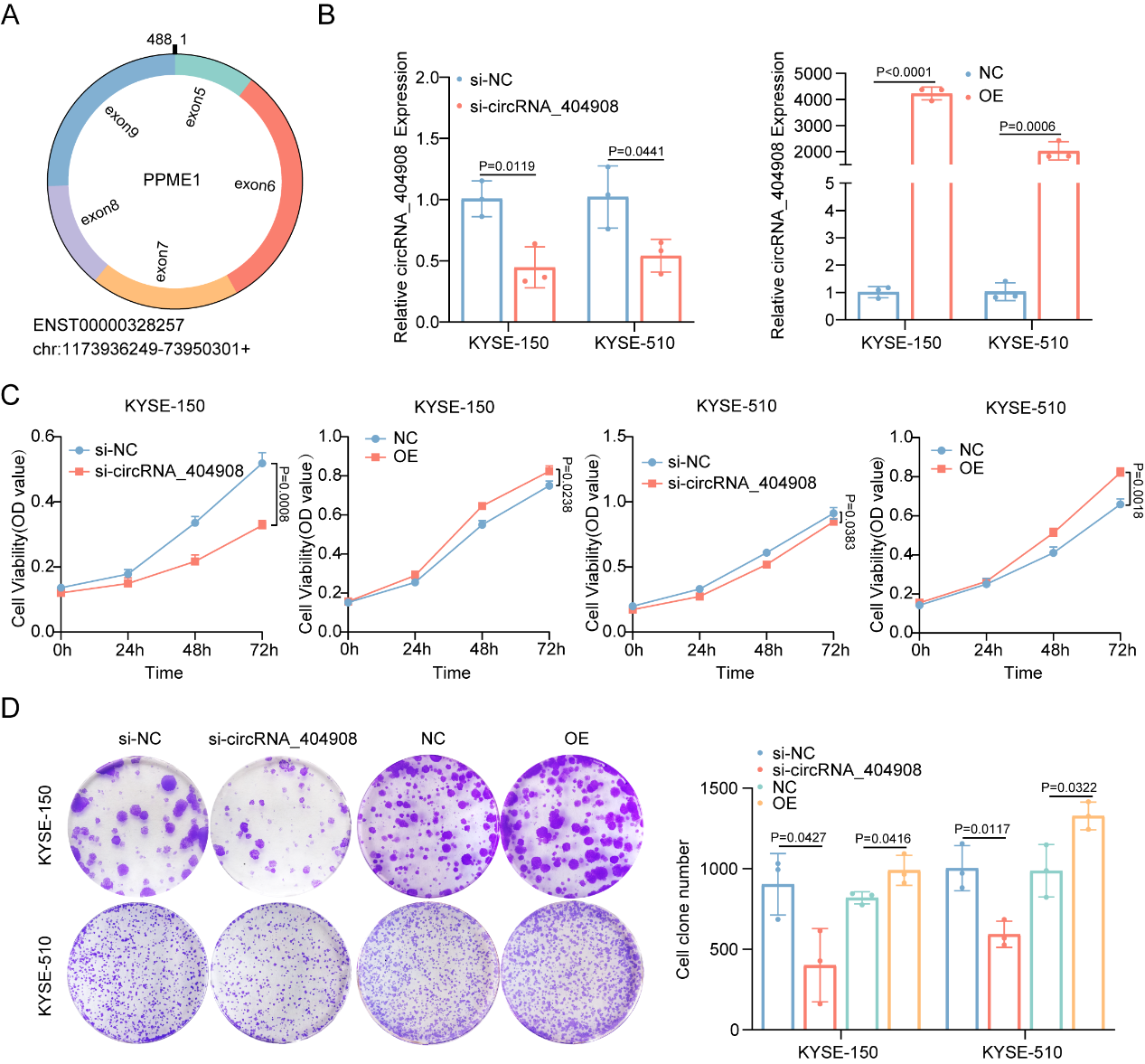


**Fig S5** The knockdown and Overexpression Efficiency Verification of circRNA_404908 and circRNA_404908 Can Promote the Proliferation Activity of ESCC Cells.

**A** Schematic representation of circRNA_4040908 circularization. **B** RT-qPCR experiments to detect the knockdown and Overexpression Efficiency of circRNA_404908. **C** and **D** CCK-8 and Plate Clone assays to detect the changes in the proliferation activity of KYSE-150 and KYSE-510 cells after Knockdown and Overexpression of circRNA_404908.


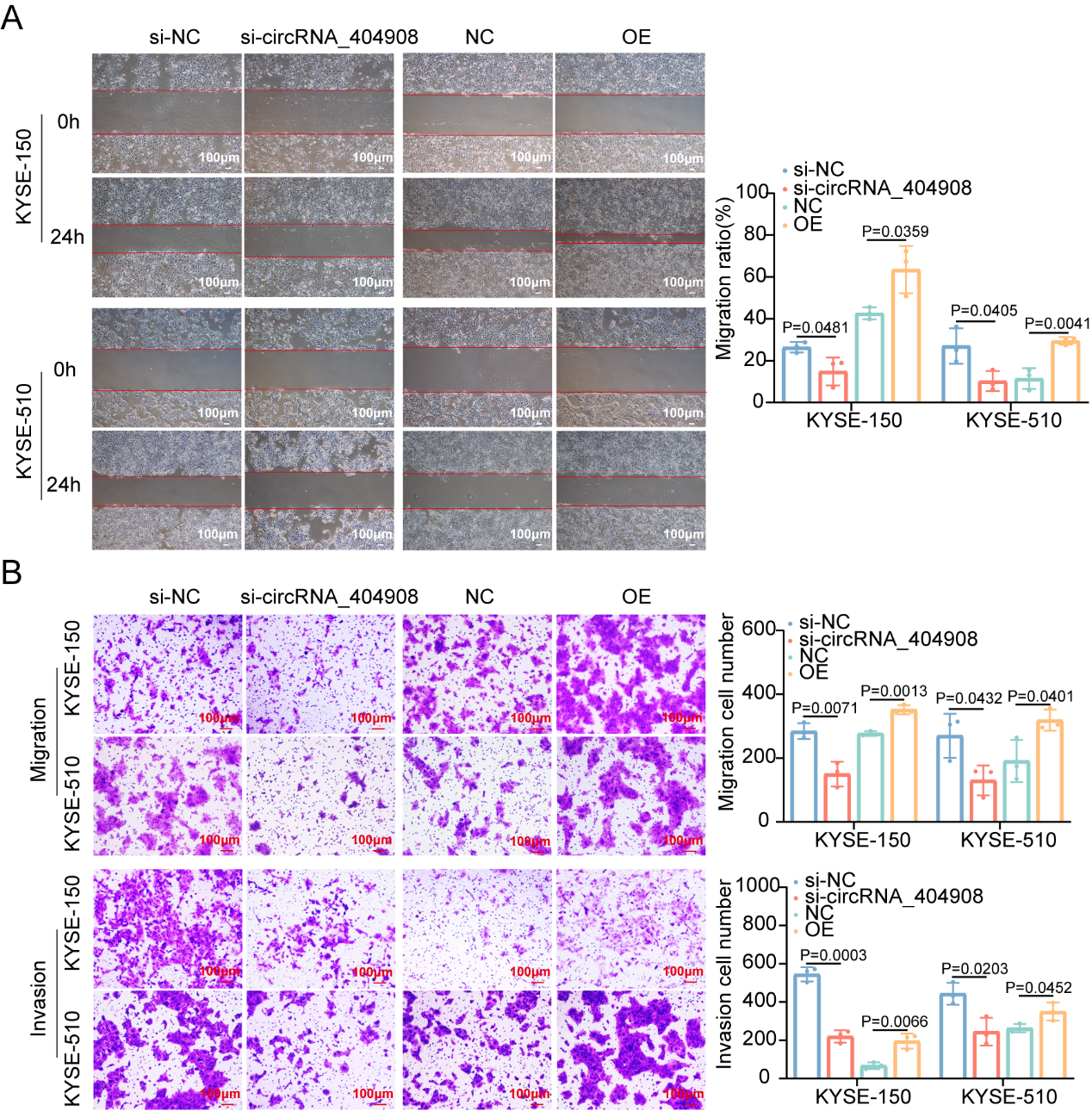


**Fig S6** circRNA_404908 can enhance the migration and invasion ability of ESCC cells.

**A** and **B** Cell scratch and Transwell assays to detect the effects of Knockdown and Overexpression of circRNA_404908 on the migration and invasion ability of KYSE-150 and KYSE-510 cells.


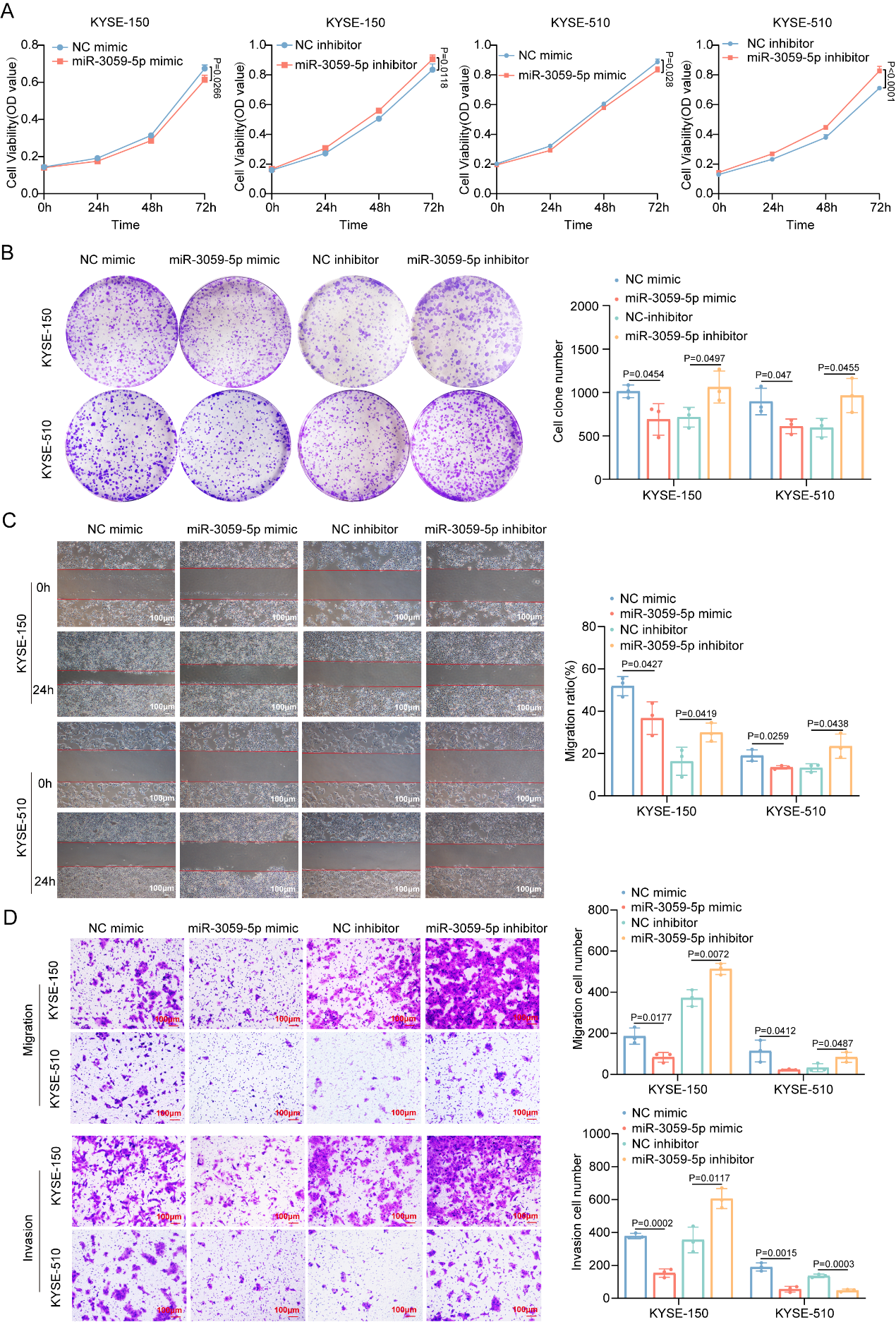


**Fig S7** miR-3059-5p has an inhibitory effect on the proliferation, migration and invasion of ESCC cells.

**A** and **B** CCK-8 and colony formation assays to detect the effects of miR-3059-5p mimic and miR-3059-5p inhibitor on the proliferation ability of ESCC cells. **C** and **D** Cell scratch and Transwell assays to detect the effects of miR-3059-5p mimic and miR-3059-5p inhibitor on the migration and invasion ability of ESCC cells.


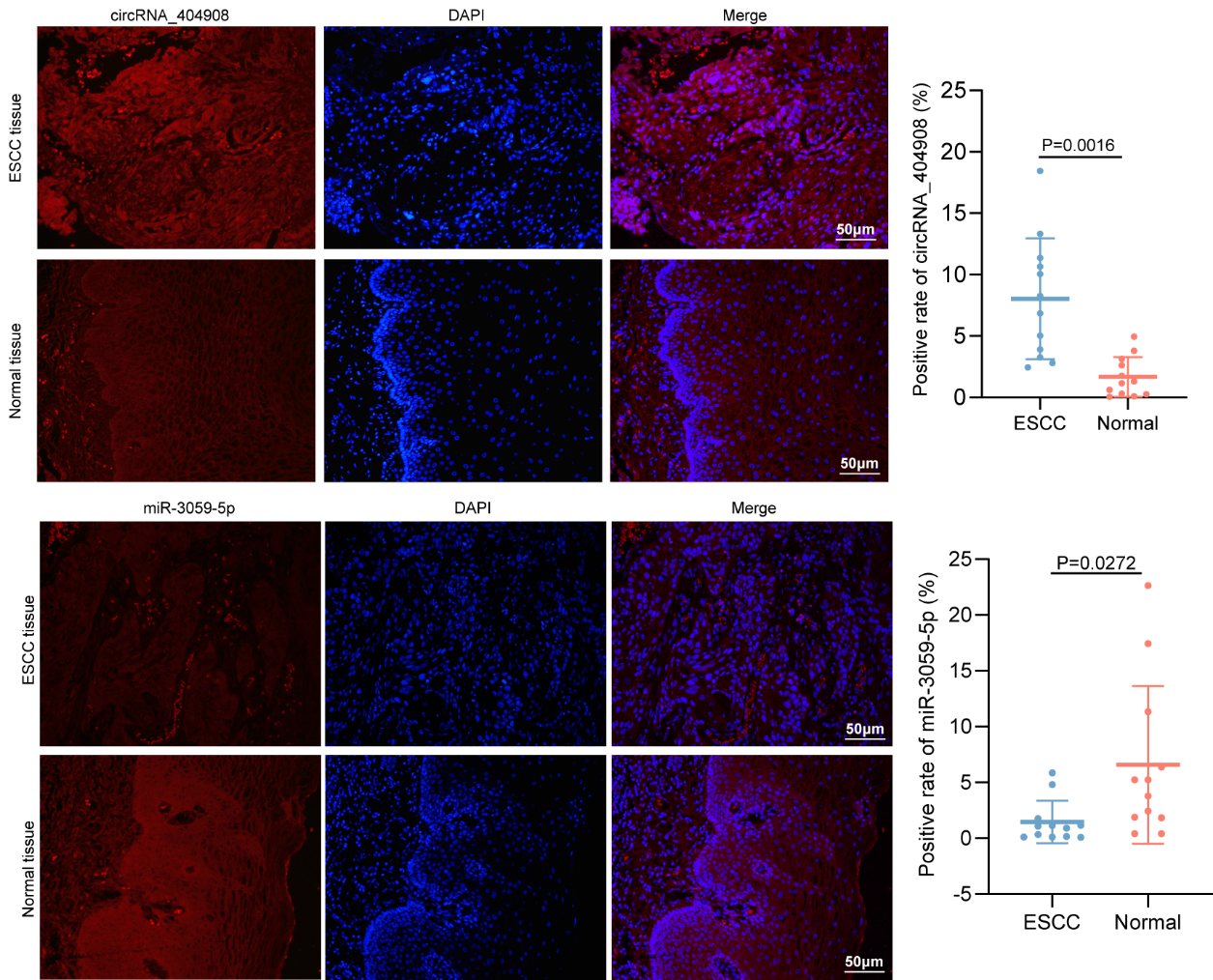


**Fig S8** Expression of circRNA_404908 and miR-3059-5p in ESCC tissue


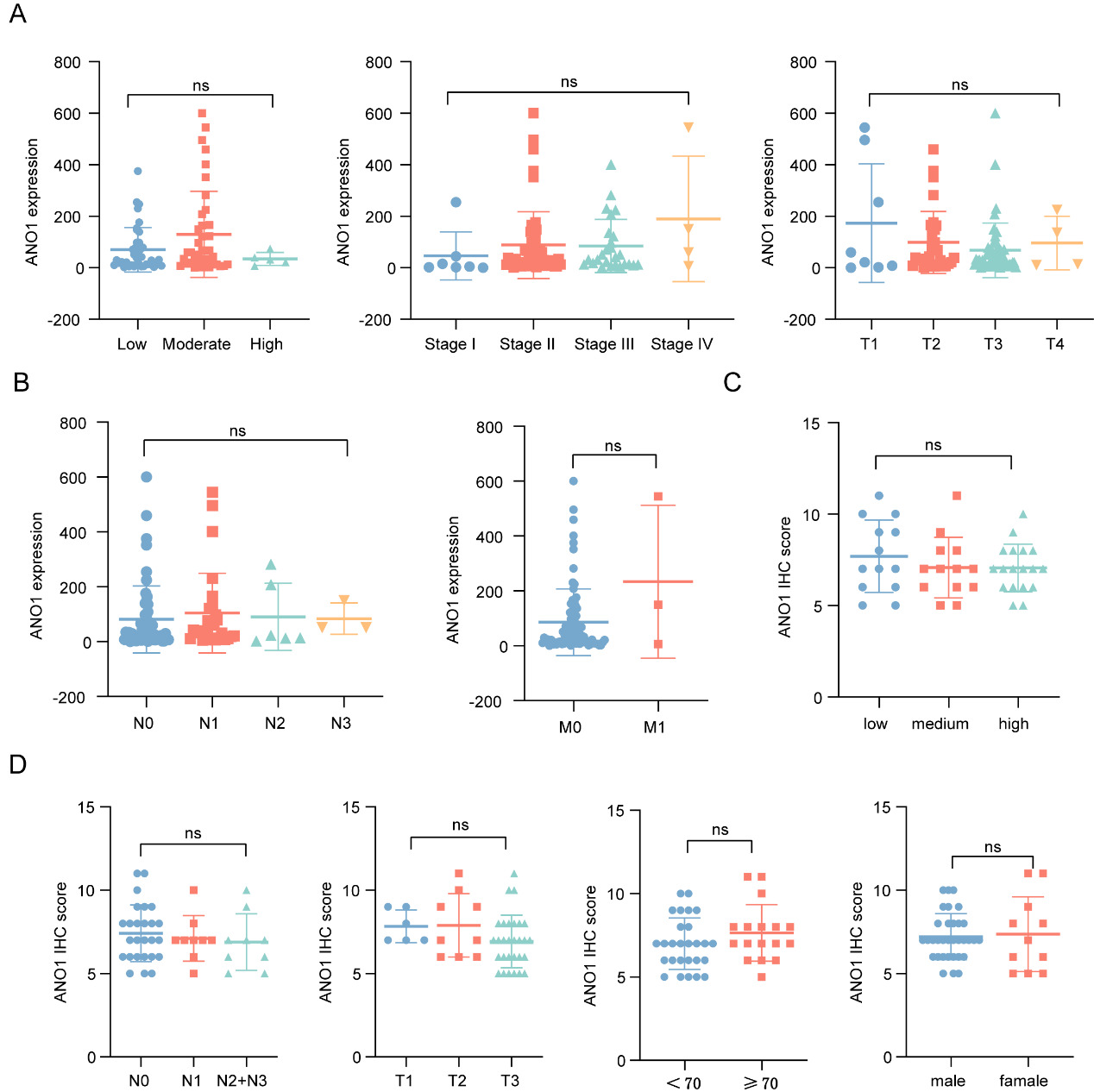


**Fig S9** The expression level of ANO1 has no significant correlation with the degree of differentiation, TNM stage, age and gender of ESCC patients.

**A** and **B** Analysis of the correlation between the expression level of WTAP in the TCGA database and the degree of differentiation, clinical stage and TNM stage of ESCC patients. **C** and **D** Analysis of the correlation between the degree of differentiation, TNM stage, age and gender of ESCC patients in immunohistochemistry and the expression score of WTAP.


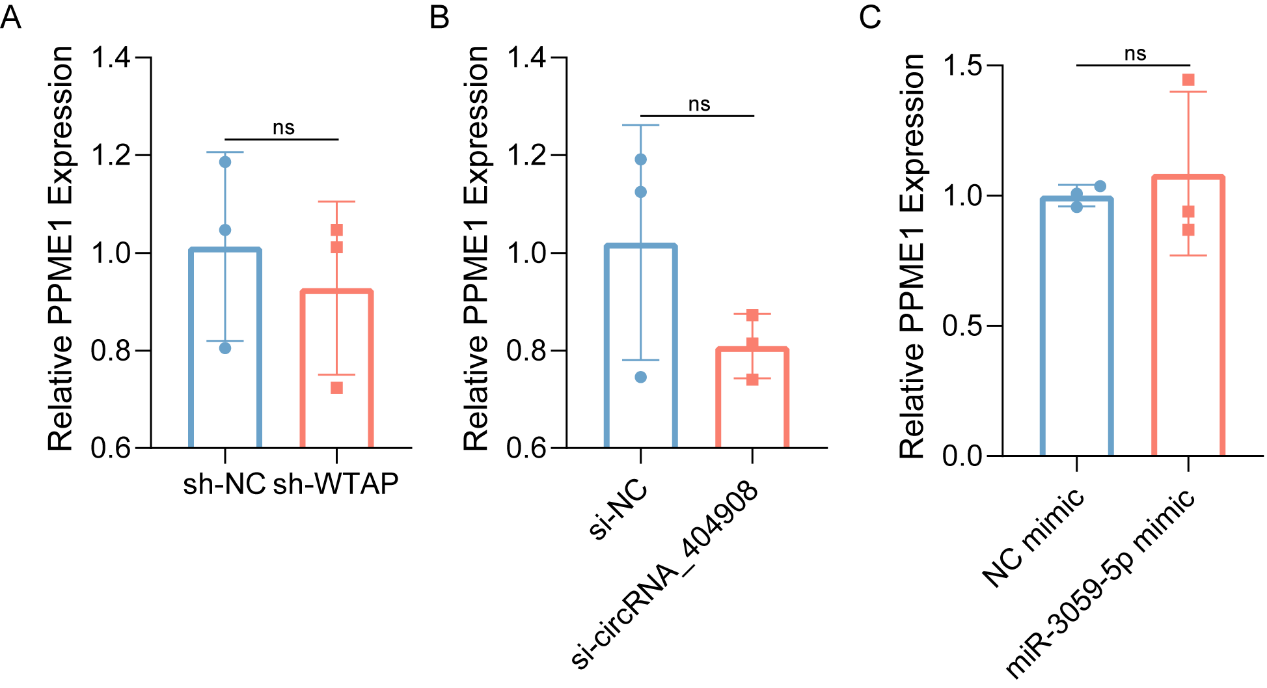


**Fig S10** Correlation of PPME1 expression level with WTAP, circRNA-404908 and miR-3059-5p

**A** Changes in PPME1 expression after knockdown of WTAP. **B** Changes in PPME1 expression after knockdown of circRNA_404908. **C** Changes in PPME1 expression after transfection of miR-3059-5p mimic
